# Variations in deep-sea methane seepage linked to millennial-scale changes in bottom water temperatures ~ 50–6 ka, NW Svalbard margin

**DOI:** 10.1038/s41598-024-72865-3

**Published:** 2024-09-27

**Authors:** Tine L. Rasmussen, Naima El bani Altuna, Erik Thomsen

**Affiliations:** 1 Department of Geosciences, UiT the Arctic University of Norway, Tromsø, 9010 Norway; 2 Department of Geosciences, Aarhus University, Aarhus, 8000 Denmark

**Keywords:** Biogeochemistry, Environmental sciences, Ocean sciences

## Abstract

During the last glaciation, the northern hemisphere experienced profound millennial-scale changes (termed Dansgaard-Oeschger (DO) events) in atmospheric and oceanic temperatures. In the North Atlantic, the fluctuations resulted in extremely unstable bottom water conditions with bottom water temperatures (BWT) varying up to > 5 °C. We have studied these changes in a core from 1,300 m water depth at Vestnesa Ridge, northwestern Svalbard margin to investigate a possible connection between BWT and seepage of methane from the seafloor covering the period ~ 50–6 ka. Beneath Vestnesa Ridge, gas hydrates containing vast amounts of methane are kept stable due to the high pressure and low temperatures. Release of gas is shown by numerous pockmarks on the seafloor. The pockmarks at 1,300 m water depth are presently inactive, but they bear witness of earlier activity. Our study shows that from ~ 50–6 ka, the core site experienced repeated increases in BWT and in the emissions of gas, both following the pattern of the DO events. This correspondence in time scale indicates that BWT was the primary forcing factor for the variability in methane release. However, the releases were delayed by up to > 1,000 years compared to the initial increase in BWT.

**Supplementary Information:**

The online version contains supplementary material available at 10.1038/s41598-024-72865-3.

## Introduction

Numerous studies have shown that the northern North Atlantic, the Nordic Seas and the Svalbard margin during the last glaciation, 50–12 ka, was affected by twelve abrupt temperature fluctuations^1–7^, which correlate closely with the millennial timescale Dansgaard-Oeschger (DO) events in the Greenland ice cores^8,9^. The DO events of cold stadials to warm interstadials reflect a series of abrupt warmings over Greenland of up to 16 °C^10^. The main cause for the DO-events is repeated reversals of the Atlantic Meridional Ocean Circulation (AMOC) and stops in deep water convection^1,4–7,11^ (see below). The abrupt changes affected both the ocean surface and bottom water temperatures (BWT). During the interstadials the ocean surface water in the Nordic Seas was warm and the bottom water cold whereas during the stadials, the surface water was cold and the bottom water warm. During the stadial periods, BWT in the Nordic Seas reached 5.5 °C at 1,200 to 1,300 m water depth^6,7^. This is close to 6 °C above present day temperatures.

These large temperature changes at the deep seafloor could have had a profound impact on the seepage of gas from dissolved gas hydrates buried on the continental slopes^12–15^. Methane, a powerful greenhouse gas, is presently released into the oceans from these thermogenic and biogenic gas reservoirs. Most of the gas is trapped in ice in the Gas Hydrate Stability Zone (GHSZ) and kept stable by the high pressure and low temperature^16^. The GHSZ on the slopes can be several hundred meters thick and hosts vast amounts of gas hydrates^17^. The highest concentrations of gas hydrates are generally located deep in the sediments depending on the presence of sediments of high porosity^18^. Facilitated by faults and porous sediments, free and dissolved gas in the pore water can migrate upward through the GHSZ and into overlying sediments with the potential to reach the atmosphere^19–22^. Growing concern about the effect of greenhouse gases on the global warming and rising surface and deep ocean temperatures and on the increasing deep-ocean acidification^14,23–26^ has fostered an intensified interest on the future stability of the GHSZ^14,15,21,22,27–30^.

Several studies have shown that there is an overall correlation between variations in BWT and the intensity of the methane leaks^21,29,31^. Paleotemperature reconstructions from deep ocean sites in the Nordic seas comprising the last glacial period are few^4–7,32,33^.

In the present study we have reconstructed temperature fluctuations in a core from 1,300 m water depth at Vestnesa Ridge on the northwestern Svalbard margin covering the time-period ~ 50–6 ka. The purpose of the study is to investigate if there is a connection between BWT and the seepage of methane from the seafloor. The locality was selected because it represents a deep-water environment affected by present and past seepage of gas and more importantly, it includes the most unstable period of the last glaciation, 50–12 ka. This permits us to examine and compare gas hydrate stability during several warming and cooling events. Furthermore, the close connection between the oceanographic fluctuations in the North Atlantic and the temperature oscillations recorded in Greenland ice cores allows us to obtain a high degree of precision in the correlation of individual events.

**Fig. 1:**
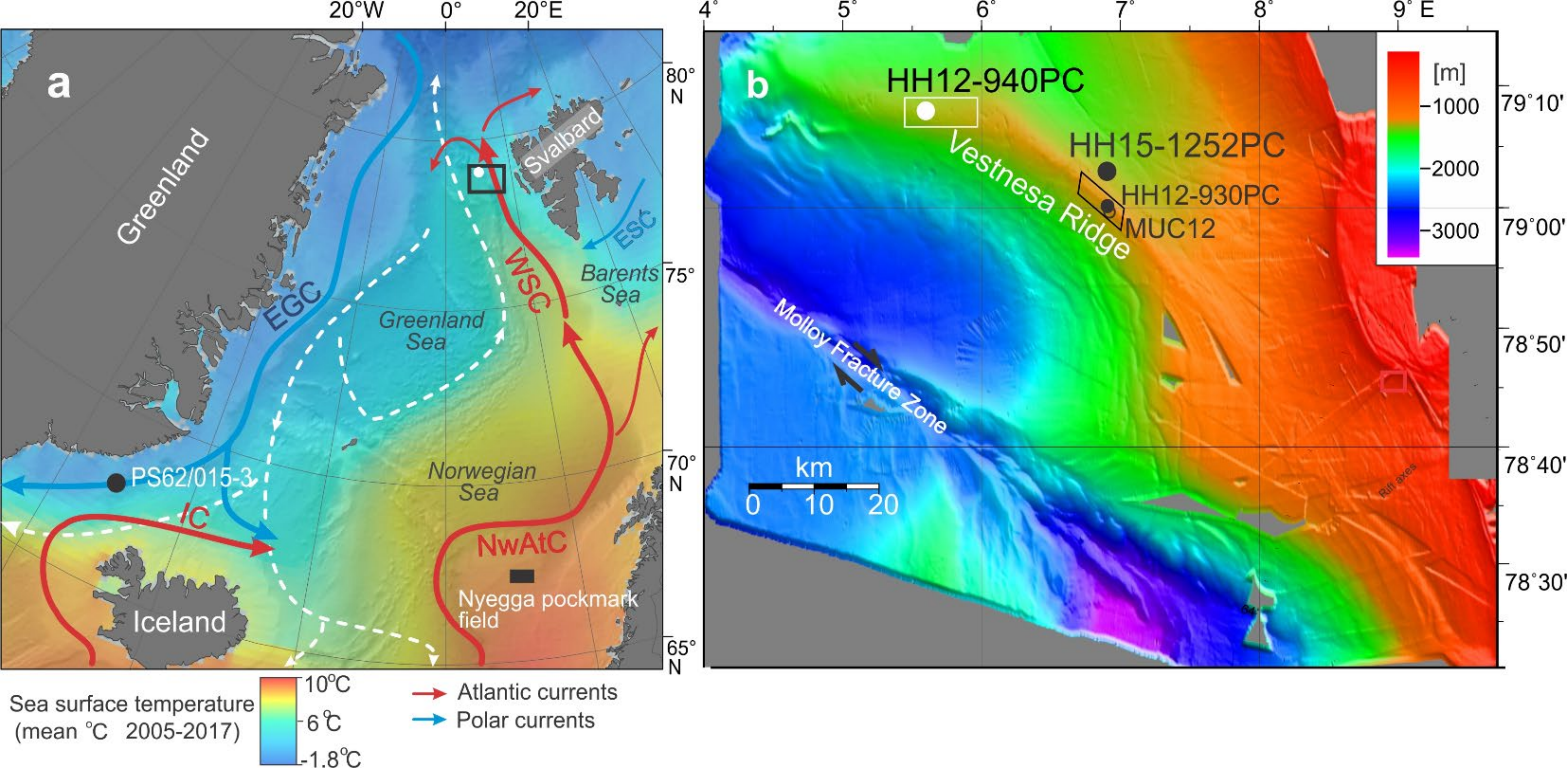
Major currents, bathymetry, and location of studied cores. **a** Map of Nordic Seas showing major currents and location of study area (black frame) and of studied core HH12-940PC (white dot). Core PS62/015 − 3 from the Denmark Strait (black dot)^32^ and the Nyegga pockmark area (black rectangle) are also indicated^33^. Coloring of map shows mean annual sea surface temperature for the 2005–2017 period^84^. The map was generated using Ocean Data View (https://odv.awi.de) with bathymetry from GEBCO (GEBCO Compilation Group (2023), GEBCO 2023 Grid (doi:10.5285/f98b053b-0cbc-6c23-e053-6c86abc0af7b). **b** Bathymetric map of western Vestnesa Ridge generated from IBCAO^85^ showing positions of core HH12-940PC (water depth 1,294 m) and of published records discussed in the text. Core HH15-1252PC are from non-seep conditions north of the ridge^7^; core HH12-930GC, and multicore MUC 12 are from eastern Vestnesa Ridge^47,52^. White frame marks the western inactive pockmark field; black frame marks the eastern active pockmark field. NwAC, Norwegian Atlantic Current; IC, Irminger Current; WSC , West Spitsbergen Current; EGC, East Greenland Current; ESC , East Spitsbergen Current.

The study is based on 230 samples from marine core HH12-940PC (Fig. 1a, b). The study comprises records of relative and absolute abundance of planktic and benthic foraminiferal species, concentration of ice rafted debris (IRD), planktic and benthic carbon and oxygen isotopes (δ^13^C and δ^18^O), and BWT calculated by transfer functions of the benthic foraminiferal census data. The detailed stratigraphy of the core is established by correlation to a nearby non-seep core HH15-1252PC from similar water depth. This core has been tied to the individual DO events 12–1 in the Greenland NGRIP ice core^7^. The reconstruction of changes in the strength of methane seepage is based on carbon isotopes combined with foraminiferal and macrofaunal census data (chemosymbiotic bivalves). Carbonate nodules from authigenic precipitation of carbonates from methane seepage were also quantified and measured for carbon and oxygen isotopes.

### Millennial-scale fluctuations in temperature and foraminiferal faunas, NW Svalbard Margin

Bottom water temperature changes on the western Svalbard margin from ~ 50 to 12 ka are closely linked to the millennial time-scale climatic and oceanographic fluctuations that characterize the North Atlantic region during the last glaciation. These fluctuations are well documented in the Greenland ice cores as well as in North Atlantic sediment cores and a precise correlation between the two systems has been established^1–9^. This allows the transfer of the names, numbering, and configuration of the DO events in the Greenland ice cores to the sediment cores of Vestnesa Ridge, including the identification of the warm interstadial and the cold stadial events^7,34^.

In the marine records the stadials are defined by low benthic and planktic δ^18^O values, the presence of IRD, and dominance of the polar planktic foraminiferal species *Neogloboquadrina pachyderma*^1^. The warm interstadials are defined by high benthic and planktic δ^18^O values, no or low IRD content, and low percentage of *N. pachyderma*^1^. Particularly cold and long lasting stadials in the marine records are termed Heinrich stadials (H). They mark prolonged IRD-events of icebergs released from the Laurentide Ice Sheet at every 6–10 ka^1^. Numerous studies of cores from the Nordic Seas and the northern North Atlantic Ocean have shown that the interstadials and stadials at mid-depth are characterized by very different foraminiferal faunas. The interstadials are dominated by the benthic species *Melonis barleeanus*, *Nonionella* spp., *Stainforthia* spp., and *Cassidulina reniforme*, while the stadials are dominated by *C. neoteretis* with the appearance of a group of Atlantic species of subtropical to boreal affinity during Heinrich stadials. This distinctive pattern makes the DO events easily recognizable in the Nordic seas especially at high latitudes^3,6,7,34–36^.

The causes for the millennial-scale fluctuations are generally attributed to instabilities in the strength and mode of the AMOC. Today, warm Atlantic Water of the Norwegian-Atlantic Current flows into the Nordic Seas at the surface (Fig. 1a). It is cooled during wintertime and convected to form cold deep and intermediate water which overflows the Greenland-Scotland Ridge contributing to North Atlantic Deep Water^11^ (Fig. 1a). During the cold periods (stadials and Heinrich stadials), the sea surface of the Nordic Seas and North Atlantic was covered by sea ice and icebergs causing stratification of the upper water column and deep convection stopped^2–7,11,37^. These surface water conditions are reflected in very low δ^18^O values. Below the cold sea surface, Atlantic water continued to flow into the North Atlantic and Nordic Seas, here warming the intermediate and subsurface water masses. The natural cold surface conditions were interrupted by abrupt warmings due to rising of the warm intermediate water to the surface. The sea-ice and icebergs melted allowing warm Atlantic water to re-enter the North Atlantic and Nordic Seas at the surface (interstadials). In the Nordic Seas, deep convection was abruptly reestablished causing a renewed cooling of the intermediate and deeper water. However, due to increasing amounts of meltwater from the surrounding ice sheets, the Nordic Seas once again became stratified, and the cold surface water and warm bottom water returned (stadials).

### Study area

Vestnesa Ridge is a southeast-northwest oriented ridge (05–08 °E) located at 79 °N at water depth between 1,200 m to the east and > 1,300 m to the west (Fig. 1b). The ridge is in direct contact with cold intermediate water (– 0.5 °C) generated by the convection in the Nordic Seas^38^. The ridge is covered by thick contourite sediments, where core HH12-940PC was taken.

The crest of the ridge is marked by a series of pockmarks which at 1,200 m water depth at the eastern part show intense seepage of methane^39–41^. The gas migrates upwards from a deep thermogenic reservoir through faulted chimneys below the pockmarks, and gas flares rise more than 800 m from the seafloor^40,42^. The western part is apparently less active as indicated by the presence of several inactive pockmarks with no visible flares^40^. On Vestnesa Ridge, the base of the GHSZ is located ~ 160–180 m below the sediment surface^41,43^. Gas hydrates have been found up to a few meters below the sediment surface in some of the active pockmarks in the eastern part^42,44,45^. The sulfate-methane transition zone (SMTZ) designating the transition of anaerobic oxidation of methane by archaea and sulfate-reducing bacteria^46^ is located > 2 to ~10 m below present sediment surface on the western inactive part indicating little or no seepage^44^.

Core HH12-940PC was taken from a sediment-filled pockmark at 1294 m water depth from the western part of Vestnesa Ridge (79.08 °N, 05.36 °E) (Fig. 1) (from here on termed core 940). This part of the ridge was active in the past, but is presently inactive^39,41,47^. To test our results, we compare the records from core 940, with records from nearby non-seep core HH15-1252PC taken north of Vestnesa Ridge^7^ (from here on termed core 1252 (Fig. 1b). Core 1252 covers the same time period as core 940. It furthermore comprises a quantification of BWT based on the Mg/Ca ratio of benthic foraminifera. The sediments at both core sites consist mainly of fine-grained hemipelagic mud and clayey silts.

## Results

### Stratigraphy and identification of Dansgaard-Oeschger events

**Fig. 2:**
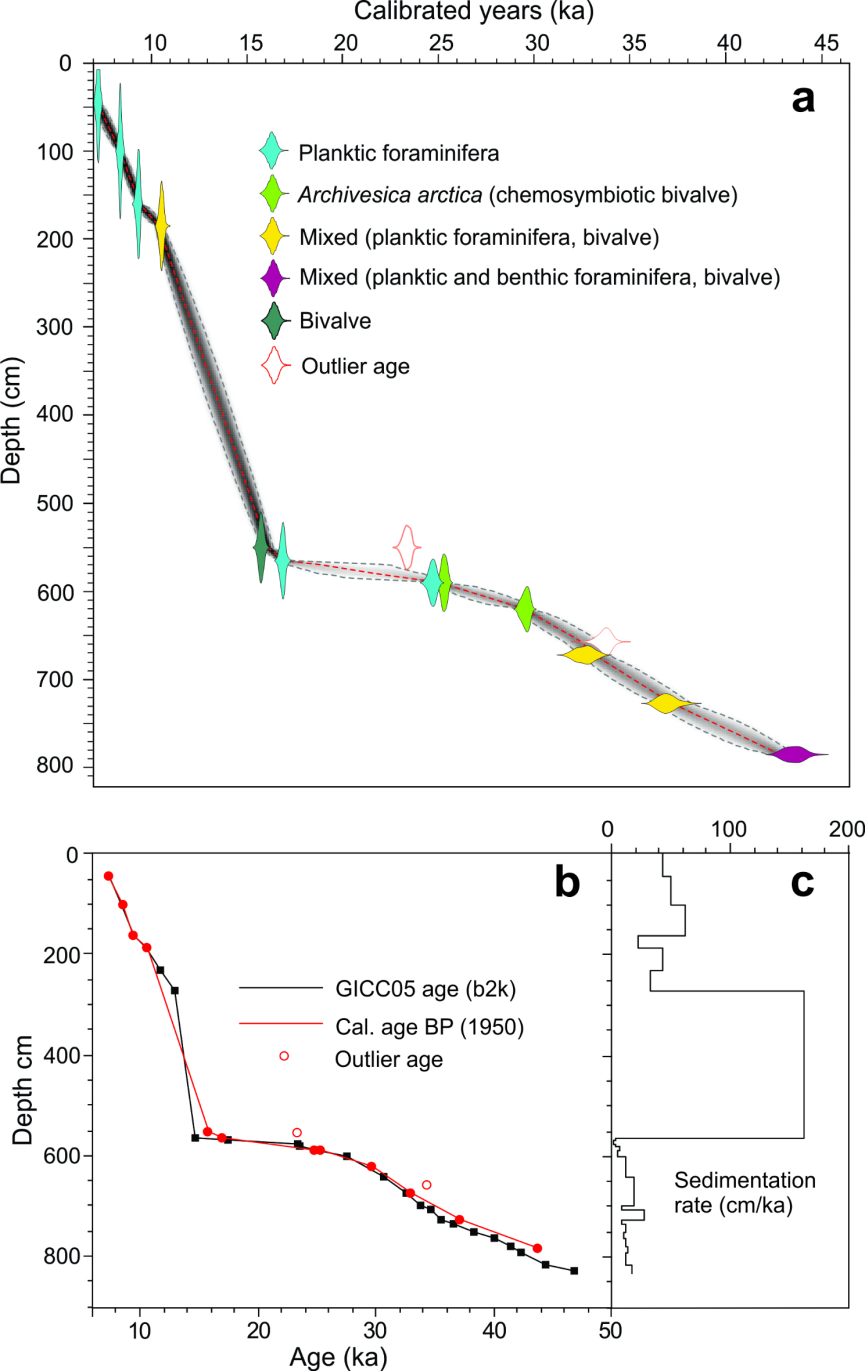
Age-depth plot, correlation to NGRIP ice core and sedimentation rates of core HH12-940PC. **a** Age-depth plot based on calibrated AMS-^14^C dates using Bayesian modeling showing probability ranges for each date (see “Methods” for explanation and Table 1). **b** Age-depth plot based on correlation with the NGRIP ice core on GICC05 time scale (b2k = before 2 ka)^8^ (black squares) plotted together with calibrated ages (red dots). Outlier dates are marked by red circles. **c** Sedimentation rates (cm/ka) calculated based on correlation points to the ice core.

**Fig. 3:**
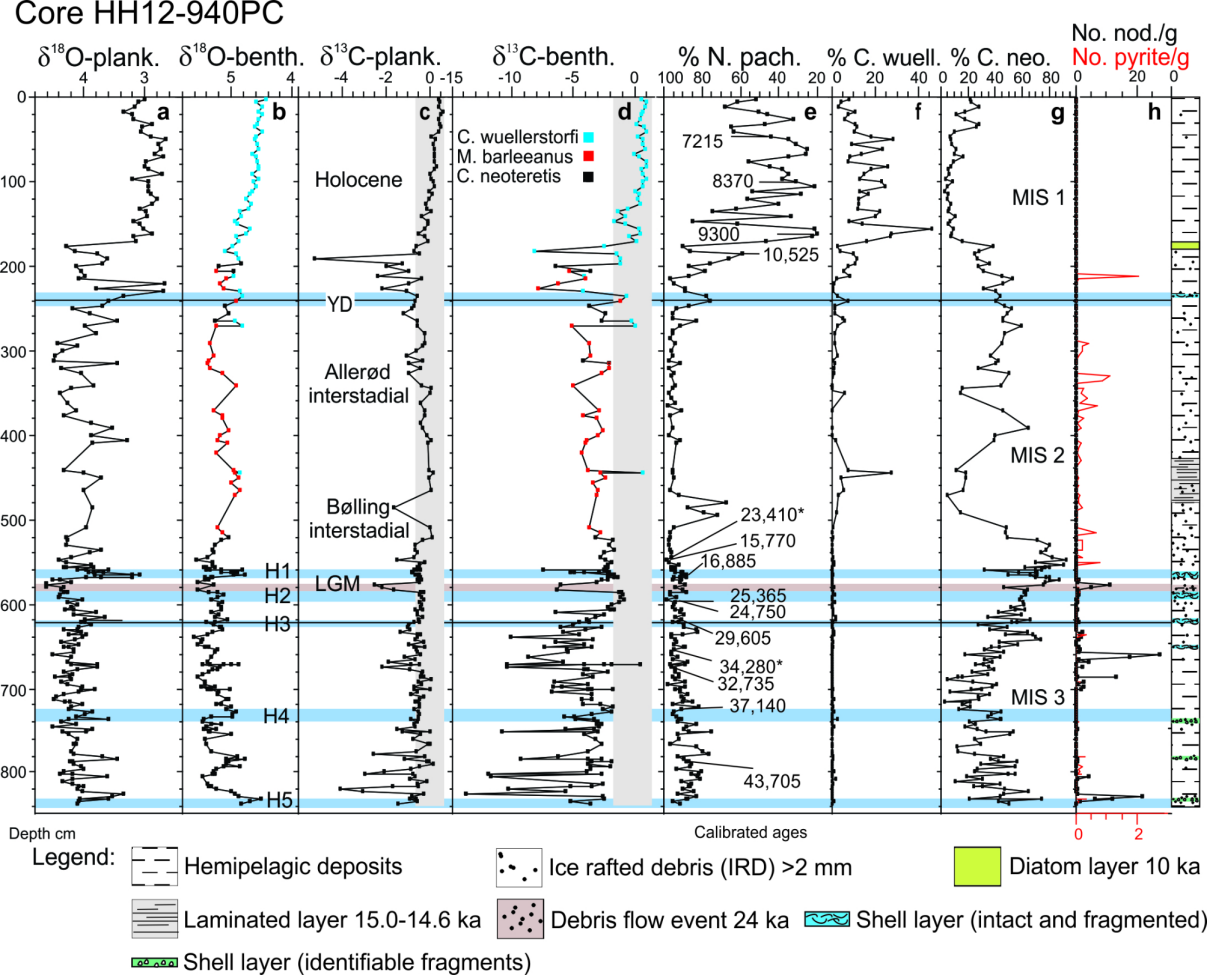
Selected records from core HH12-940PC. **a** Planktic δ^18^O measured in the foraminiferal species *Neogloboquadrina pachyderma*. **b** Corrected (see “Methods”) benthic δ^18^O measured on *Cibicidoides wuellerstorfi* (blue squares), *Melonis barleeanus* (red squares) and *Cassidulina neoteretis* (black squares). **c** Planktic δ^13^C measured in *N. pachyderma*. Vertical grey bar indicates typical range of glacial and interglacial values. **d** Benthic δ^13^C measured in *C. wuellerstorfi* (blue squares), *M. barleeanus* (red squares) and *C. neoteretis* (black squares). Vertical grey bar indicates typical range of glacial and interglacial values (see text for explanation). **e** Percentage distribution of polar planktic foraminiferal species *N. pachyderma* (note inverse scale) with position of calibrated AMS-^14^C dates indicated, asterisks mark outlier dates (see Table 1). **f** Percentage of epifaunal benthic species *C. wuellerstorfi*. **g** Percentage of shallow infaunal benthic species *C. neoteretis*. **h** Concentration of calcareous nodules > 0.5 mm from authigenic carbonate precipitation (see text for explanation) in number per gram dry weight sediment (black curve) and concentration of pyrite particles > 0.5 mm in number per gram dry weight sediment (red curve). Corelog shown to the right of (**h**). YD ,Younger Dryas; LGM, Last Glacial Maximum; H , Heinrich stadial; MIS, Marine Isotope Stage. Blue bars mark Heinrich stadials, horizontal black lines isotope stage boundaries.

Twelve out of 14 AMS-^14^C dates are in chronological order (Fig. 2; Table 1; Figs. S1, S2). The two outlier dates are older than the dates above and could have been affected by old carbonate from the seepage of gas^48^. The AMS-^14^C dates in combination with the δ^18^O record indicate that the core comprises the mid-late part of marine isotope stage (MIS) 3, the whole of MIS 2 and the early part of MIS 1 (mid-late Weichselian to mid Holocene) (Fig. 3a, b). Based on the lowermost two calibrated dates and assuming linear sedimentation rate, the oldest sediments of the core are calculated to ~ 50 ka (48 ka in ice core years; Fig. 2). Similarly, from the uppermost two calibrated dates, the youngest sediments are calculated to ~ 6.3 ka.

**Table 1:** AMS-^14^C dates and calibrated ages for core HH12-940PC from Vestnesa Ridge, northwest Svalbard.

Depth cm	^14^C -age	Lab. code	Species	ΔR (σ1 years)	Calibrated age ranges (1σ error)	Average calibrated age (1σ error)
45.5	6833 ± 30	UB31813	*N. pachyderma*	– 65 ± 33	[7380, 7050]	7215 ± 165
100.5	8012 ± 30	UB31814	*N. pachyderma*	– 65 ± 33	[8520, 8220]	8370 ± 150
160.5	8754 ± 32	UB31815	Mixed PF.	– 65 ± 33	[9460, 9140]	9300 ± 160
185.5	9710 ± 43	UB31852	*Thyasira*, Mixed PF.	– 65 ± 33	[10740, 10310]	10525 ± 215
550.5	20184 ± 99	UB31816	*N. pachyderma*	– 65 ± 33	[23720, 23100]	23410 ± 310
550.5	13688 ± 50	UB31853	*Thyasira* sp.	– 65 ± 33	[16020, 15520]	15770±250
565.5	14577 ± 52	UB31817	*N. pachyderma*	– 65 ± 33	[17140, 16630]	16885 ± 255
590.5	21899 ± 93	UB31818	*N. pachyderma*	– 65 ± 33	[25650, 25080]	25365±285
590.5	21371 ± 124	UB31854	Fr. *A. arctica*	– 65 ± 33	[25130, 24370]	24750 ± 380
620.5	26207 ± 164	UB42878	Fr. *A. arctica*	– 65 ± 33	[29980, 29230]	29605 ± 375
657.5	30085 ± 267	UB42880	*Thyasira*+*Frigido*.	– 65 ± 33	[35330, 33230]	34280 ± 1050
672.5	29212 ± 347	UB31855	*Nucula*, Nodos., *N. pach*.	– 65 ± 33	[33660, 31810]	32735 ± 925
727.5	33228 ± 366	UB42881	*Nucula solidula*	– 65 ± 33	[38110, 36170]	37140 ± 970
785.5	41568 ± 709	UB31856	*Thyasira*, PF, BF	– 65 ± 33	[44710, 42700]	43705 ± 1005

PF, planktic foraminifera; BF, benthic foraminifera; Fr., fragments; Nodos., Nodosariidae; Frigido., Frigidoalvania sp.; N. pach., N. pachyderma.

**Fig. 4:**
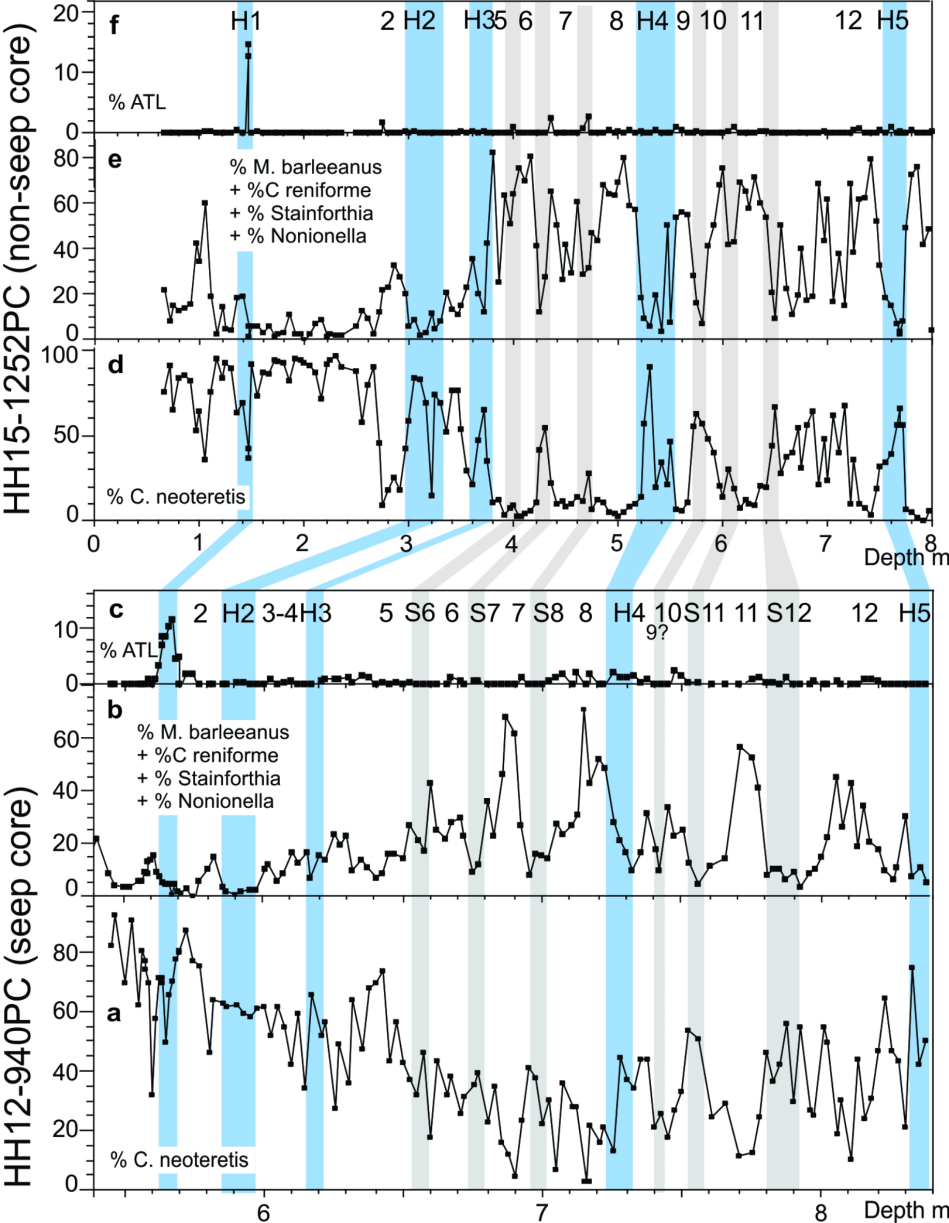
Distribution patterns of selected benthic foraminiferal species in core HH12-940PC for the lower 5.45–8.38 m and core HH15-1252PC for the interval 0–8 m (MIS 3 and MIS 2). **a** Percentage of stadial benthic foraminiferal species *Cassidulina neoteretis*. **b** Percentage of interstadial benthic species *Melonis barleeanus*., *Nonionella* spp., *Stainforthia loeblichi*, and *Cassidulina reniforme* added together (see text for explanation). **c** Percentage of Atlantic species group (ATL). Heinrich events H5 to H1 and interstadial numbers 12–2 are indicated. **d–f** Distribution of same species in core HH15-1252PC^7^. Heinrich stadials H5 to H1 and stadial (S) and interstadial numbers 12–2 are indicated. Blue horizontal bars mark Heinrich stadials, grey bars mark stadials.

**Fig. 5:**
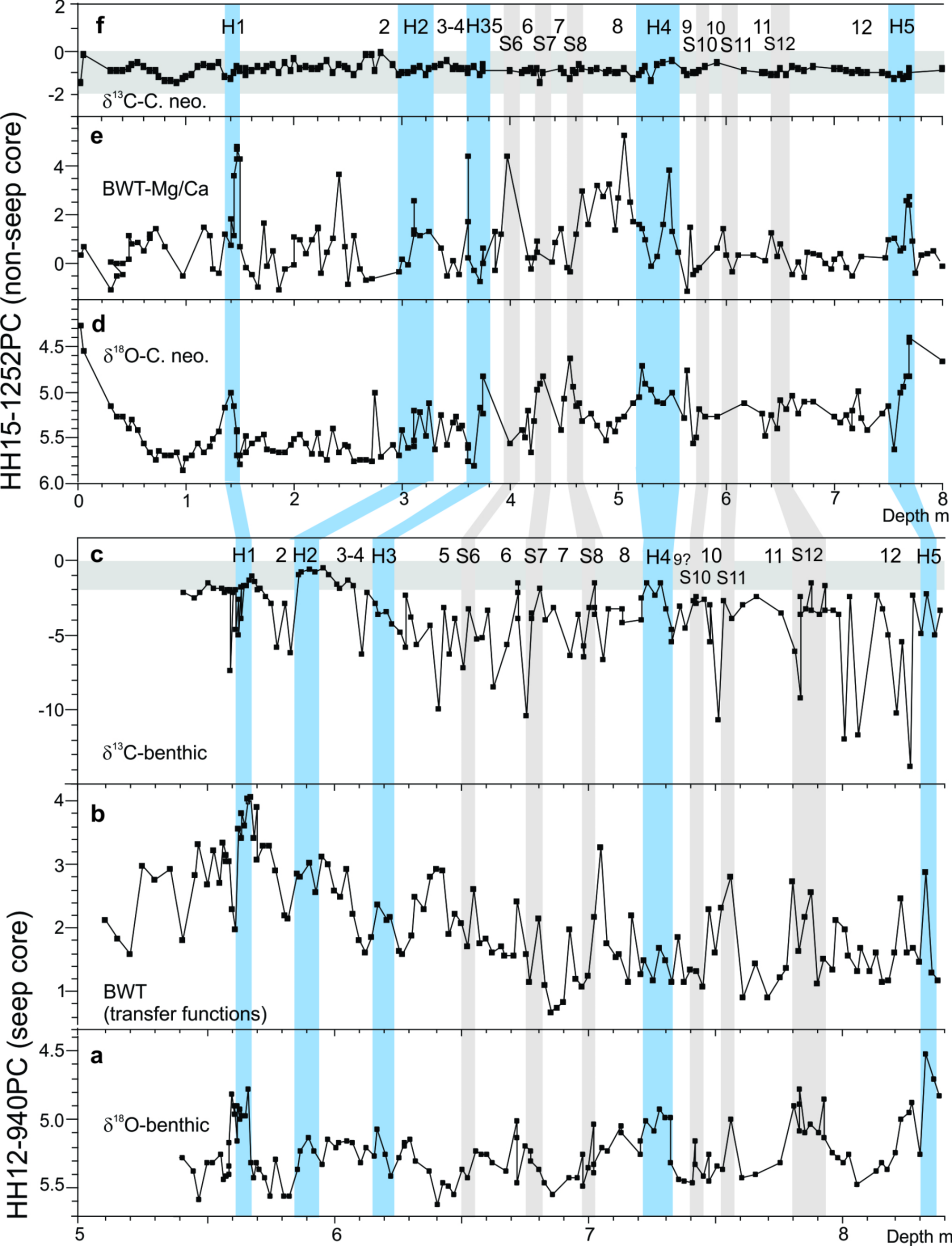
Correlation of HH12-940PC (a–c) with HH15-1252PC^7^ (d–f) for MIS 3 and MIS 2. **a** Benthic δ^18^O measured on *Melonis barleeanus* and *Cassidulina neoteretis*. **b** Bottom water temperatures (BWT) calculated from transfer functions of the benthic foraminiferal faunas (RMSEP is ± 1.8767 °C). **c** Benthic δ^13^C measured in *M. barleeanus* and *C. neoteretis*. **d** Benthic δ^18^O measured on *Cassidulina neoteretis*. **e** Bottom water temperatures (BWT) from measurements of Mg/Ca (estimated total of analytical and calibration errors are ± 1.03 °C and ± 1.27 °C for *C. neoteretis* and *M. barleeanus*, respectively^7^). **f** Benthic δ^13^C measured in *C. neoteretis*. Heinrich stadials H5 to H1 and stadial (S) and interstadial numbers 12–2 are indicated. Blue horizontal bars mark Heinrich stadials, grey bars mark stadials.

With eight species of planktic and more than 100 species of benthic foraminifera (Table S1), the overall diversity of the benthic foraminiferal faunas is high (Figs. 3, 4 and 5). The lower part of core 940 from 838 to 630 cm is referred to MIS 3 (Fig. 3). This interval shows high variability of most parameters including stable isotopes (δ^18^O and δ^13^C), BWT, the percentages of the dominating planktic and benthic species *N. pachyderma*, *C. neoteretis*, *M. barleeanus*, and *C. reniforme* (Figs. 3, 4 and 5; Figs. S2–S4; see also Supplementary text). A similar strongly fluctuating pattern is seen in non-seep core 1252 north of Vestnesa Ridge (Figs. 4 and 5; Fig. S3).

Stadials and interstadials and the boundaries between them are recognized on the basis of the distribution of benthic foraminifera following the general pattern for the Nordic seas described above. Stadials are identified by the dominance of *C. neoteretis* and/or the Atlantic Species Group. Interstadials are identified by the dominance of *M. barleeanus*, *C. reniforme*, *Nonionella* spp., and *Stainforthia* spp. (Fig. 4; Fig. S3). In Fig. 4, we have added *M. barleeanus*, *C. reniforme*, *Nonionella* spp., and *Stainforthi*a ssp. together to emphasize the contrasting shifts between the stadial and interstadial faunas (see Fig. S3 for distribution of the individual interstadial benthic species).

In the surface environment, stadials show high relative abundance of the polar planktic species *N. pachyderma*, presence of IRD, and generally low δ^18^O values. Interstadials show lower percentages of *N. pachyderma*, low or no IRD, and generally high δ^18^O (Fig. 5; Fig. S4). The BWT during MIS 3 shifts between low/lower temperatures during the interstadials and high temperatures during the stadials (Fig. 5b; Fig. S4).

The variability encountered in core 940 is characteristic for mid-late MIS 3 and MIS 2 in the Nordic Seas and North Atlantic and it allows us to identify Heinrich stadials H5, H4 and H3 as well as DO events 12–5 of MIS 3^1–10,34^. Our identification of specific DO events is confirmed by correlation of core 940 to the nearby non-seep core 1252, which was tied to the δ^18^O record of the NGRIP ice core^7^ where these events are defined and dated^8^ (Figs. 4 and 5; Fig. S3) (see “Methods”).

The early part of MIS 2 is very compressed with low sedimentation rates (Fig. 2c). We can, nevertheless, recognize interstadials 4, 3 and 2, plus H2, the last glacial maximum (LGM) and H1 (Figs. 2, 3, 4 and 5). The LGM comprises the core section from 585.5 to 570.5 cm, where it is recognized by the very high planktic and benthic δ^18^O values dated to ~ 24.0–17.5 ka in ice cores^8^ (Fig. 2a, b) correlating well with the global isotope stack record^49^. The LGM is followed by H1 from 570.5 to 560.5 cm marking the beginning of the deglaciation dated to ~ 17.5–14.7 ka^8^ (Fig. 2a, b). It is characterized by minima in the benthic and planktic δ^18^O values and a high relative abundance of the Atlantic Species Group (> 12%) (Figs. 4a and f and 5a and d). The low δ^18^O values and the occurrence of the Atlantic Species Group is typical for H1 in the North Atlantic, Nordic Seas and Arctic Ocean^3,6,7,34,36^.

The warm interstadials Bølling and Allerød 560.5–270.5 cm and dated to 14.7 to ~13.0 ka^8^ (Figs. 2a and b and 3), show an expanded section and very high sedimentation rates with an average of 160 cm/ka as compared to 2 cm/ka for the LGM and H1 (Fig. 2c). The Bølling and Allerød section appears quite disturbed with vertical channels/fractures (Fig. S1; see “Discussion”). The Younger Dryas cold stadial from 270.5 to 225.5 cm and dated to ~ 13.0 to 11.7 ka^8^ is distinguished by low planktic and benthic δ^18^O values (Figs. 2 and 3a and b). The Holocene interval from 225 cm to the core top and dated to 11.7–6.3 ka (Fig. 2) is first of all distinguished by low percentages of the polar planktic species *N. pachyderma* and low planktic and benthic δ^18^O values (Fig. 3a, b, e), and high concentrations of planktic and benthic foraminifera (Fig. S2d, e). The benthic foraminifera *C. wuellerstorfi* is relative abundant in the Holocene part (Fig. 3f).

### δ13C and temperature variability in cores 940 and 1252

The δ^13^C values of marine organisms are strongly affected by methane in the environment. Tests of the majority of intermediate infaunal (*M. barleeanus*) to shallow infaunal (*C. neoteretis*) benthic foraminiferal species living in non-seep environments show interglacial values of δ^13^C between − 1 and 0‰^48^ and glacial values between − 2 and 0‰^7^. The epifaunal species *C. wuellerstorfi* records glacial and interglacial values of 0 to > + 1‰^50^ (Fig. 3d). Values lower than about − 2‰ are usually taken to indicate the presence of methane assuming that these foraminifera would obtain the low δ^13^C values either from the surrounding pore water or from consumed food^51–53^. In the following descriptions, the − 2 to 0‰ interval is termed the typical glacial-interglacial range for the infaunal species *C. neoteretis* and *M. barleeanus* combined and used as a visual guideline in the presentation (grey vertical bar in Fig. 4c, f).

**Fig. 6:**
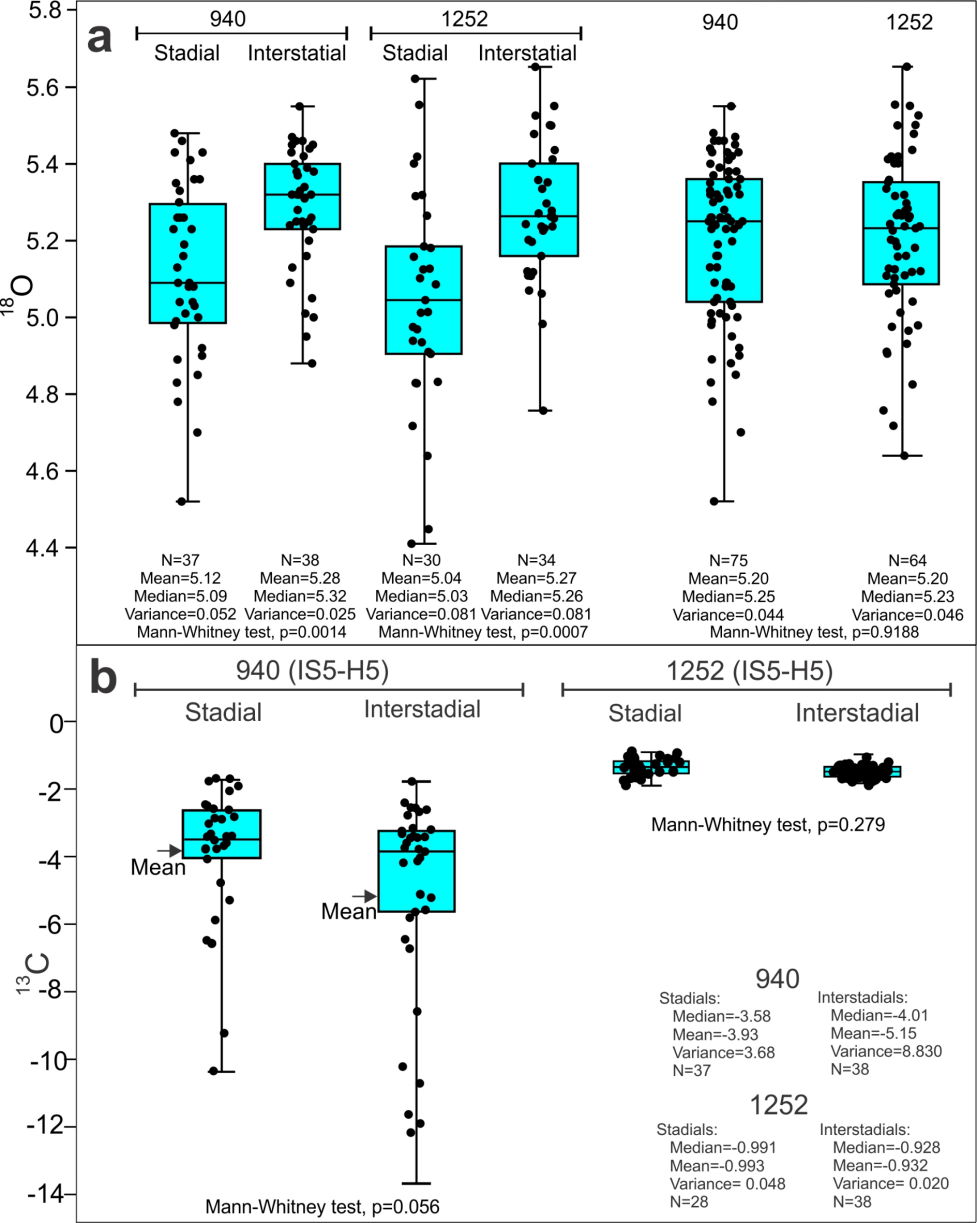
Boxplots (box and whisker plot) comparing δ^18^O and δ^13^C values from stadials and interstadials of cores 940 and 1252 comprising the interval from interstadial IS5 to Heinrich events H5 (see Fig. 5). The box represents the interquartile range of the data. The median value is indicated by a bar. The length of the whiskers indicates the range of the measured values. **a** Comparison of δ^18^O values from stadials and interstadials of cores 940 and 1252 and a comparison of all values from the two cores from the interval given above. **b** Comparison of δ^13^C values from stadials and interstadials of cores 940 and 1252 comprising the interval given above. Basic statistical information is given for each of the investigated elements. Similarities between stadial and interstadials were tested using Mann-Whitney test. The p-value for each comparison is indicated. Boxplot and tests were made using the Past3 Statistical Program^80^.

Comparing the results of core 940 with previously published data from the non-seep core 1252 covering the interval ~ 50 to 12 ka, we find the same distribution of benthic and planktic δ^18^O, the same bottom water temperatures, and the same relative abundances of benthic foraminiferal species^7^ (Figs. 4, 5 and 6a; Figs. S3, S4). This allows a close correlation of the two records comprising H1–H5 and DO events 12–2. However. with respect to δ^13^C the two sites are very different (Fig. 6b). In contrast to the fluctuating values of core 940, all δ^13^C values of core 1252 are within the typical range of – 2 to 0‰ confirming that site 1252 was without any traces of methane seepage^7^ (Figs. 5c and f and 6b). The lack of methane is further supported by the absence of carbonate nodules and chemosymbiotic mollusks, both common in core 940 (Fig. 3h; Fig. S4; see “Discussion”).

The lowest benthic δ^13^C values occur close to the stadial/interstadial boundaries - mostly at the beginning of the interstadials but sometimes at the end of the stadials (e.g., top of H5, S12 and H1; Fig. 5c). From this low point the values increase during the interstadial, but they generally remain well below the typical range of – 2 to 0‰ (Fig. 6b). At the stadial boundary the values increase further and the values of the stadials are usually higher than the interstadial values and mostly above − 4‰ (Figs. 5c and 6b). The Younger Dryas stadial show values above − 2‰ (Fig. 3d). The planktic δ^13^C record of core 940 shows low values at the beginning of many interstadials, but not as consistently as in the benthic records. Moreover, it appears that several of the planktic minima occur slightly later than the benthic minima and are of much lower magnitude (Fig. 3a–d).

In core 940 many of the interstadials (though not all) and some stadials display a high concentration of carbonate nodules (Fig. 3h; Fig. S4c). These nodules are characterized by very low δ^13^C values (– 47.2 to – 30.6‰; average − 41‰) and high δ^18^O values (5.7 to 7.2‰; average 6.7‰) (Fig. S4d, e). In core 940, Heinrich stadials and some stadials contain layers rich in chemosymbiotic bivalves and other seep-associated macrofaunas. In addition to higher δ^13^C (> – 4 to – 3‰), these faunas co-occur with low benthic δ^18^O and higher BWT (Figs. 3, 5b and c and 6b; Figs. S2, S4b, c). They are found in relatively thin discrete layers in stadials S12 and S6 and in H5, H4, H3, H2, and H1 (Fig. 3; Fig. S2﻿). The chemosymbiotic faunas are absent from the interstadials just as they are absent from the non-seep core 1252^7^.

**Fig. 7:**
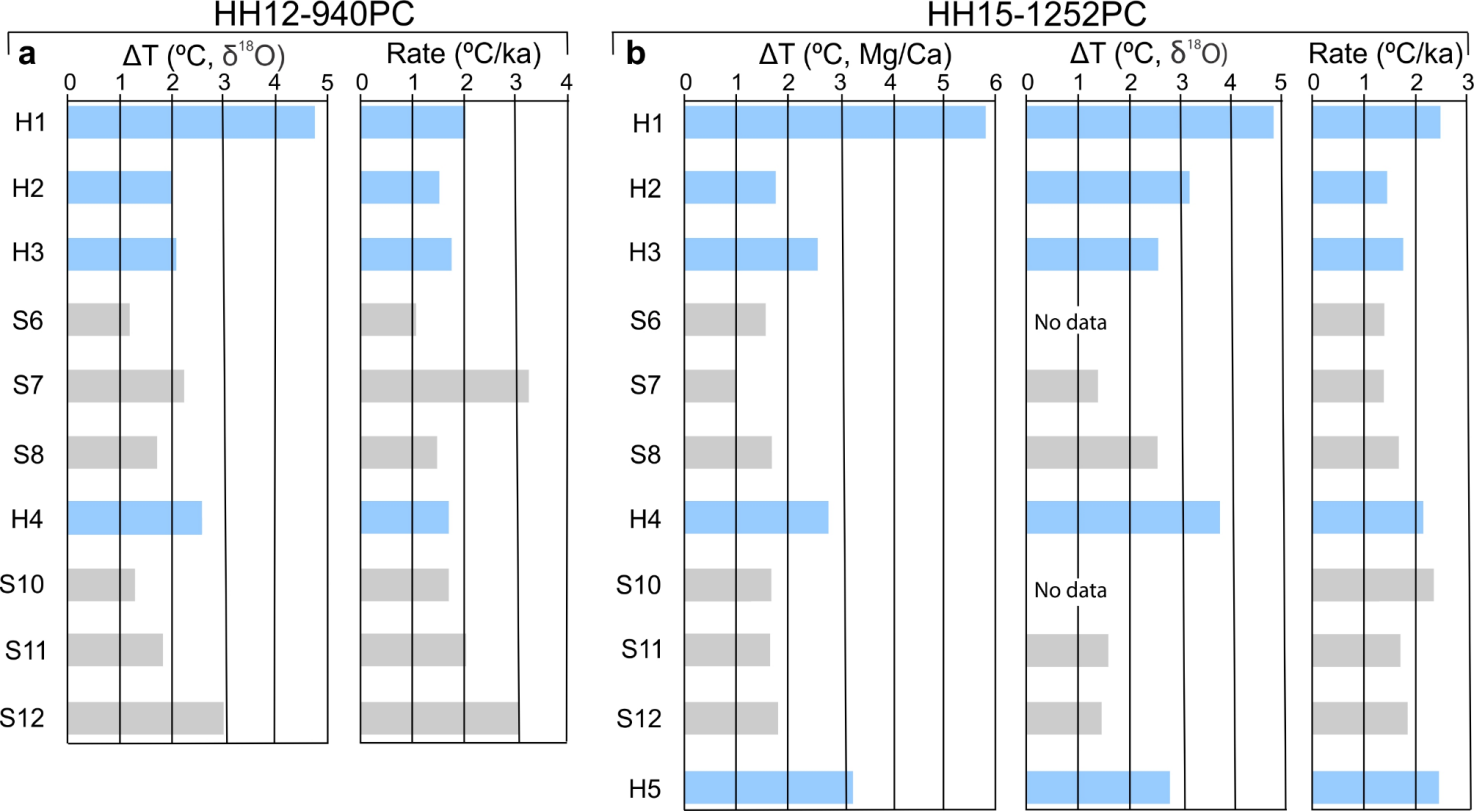
Histogram plot of bottom water temperature increases and rates. Temperature increases calculated from the end of an interstadial to the maximum obtained in the subsequent stadial/Heinrich stadial for cores HH12-940PC (left) and HH15-1252PC (right) and the calculated rate of the temperature increases are shown. Heinrich stadials H5 to H1 are indicated by blue bars and stadials (S) by grey bars.

In cores 940 and 1252, calculated average BWT shows the largest temperature rises in connection with the long-lasting Heinrich stadials (Fig. 7a, b). The temperatures generally decrease successively in the subsequent shorter-lasting stadials until the next Heinrich stadial. The calculated temperatures represent the maximum difference from the lowest temperature during the interstadials to the highest temperature during the subsequent stadial. The average rate of the temperature increases is 2.0 °C/ka (range 1.1–3.2 °C/ka) for core 940 and 1.9 °C/ka (range 1.4–2.5 °C/ka) for core 1252 (Fig. 7a, b).

## Discussion

### Dansgaard-Oeschger events and the emission of methane

The consistent millennial time-scale pattern shown by the δ^13^C events in core 940 strongly indicates that the events are not randomly distributed and that they are caused by a common forcing factor which varies concurrent with the DO oscillations. The most likely factor on the Vestnesa Ridge that fulfils these criteria is bottom water temperature which in the North Atlantic and Nordic Seas fluctuates according to the DO events^3,5–7,34,36^. However, other factors such as hydrostatic pressure/tectonism/isostatic rebound are also briefly discussed.

Glacial and interglacial δ^13^C values below the typical ranges of benthic foraminiferal species have generally been used to indicate the presence of methane^51,53^. The immediate interpretation of the variation of δ^13^C in core 940 is that the seepage of methane was low to moderate during the stadials where the δ^13^C values generally are above ~ – 4‰ (Figs. 5c and 6b). An abrupt decrease in δ^13^C close to the stadial/interstadial transitions suggests that the seepage of methane at the beginning of the interstadials increased significantly. The peak was short-lasting, but the emission remained relatively high during the rest of the interstadials (Fig. 5c). Note that after ~ 10 ka in the Holocene, high δ^13^C values in core 940 indicate a reduction or stop in seepage (Fig. 3c, d). Overall, it is important to note that the δ^13^C values in core 940 are consistently lower than in non-seep core 1252 indicating that site 940 constantly was affected by methane also during the stadials (Fig. 5; Fig. S4).

The δ^13^C values of live benthic foraminiferal tests that have been exposed to methane generally range from 0 to – 6‰, depending on species, indicating that the effect of methane on the δ^13^C in shells of foraminifera is relatively small^51,52,54,55^. This is especially evident when compared to the much lower values (– 10‰ to < – 20‰) measured in foraminiferal tests affected by authigenic carbonate^5,42,57^. These coatings, which usually are precipitated on dead tests, are particularly common in methane affected environments, where the precipitation is a result of anaerobic oxidation of methane with sulfate reduction in the SMTZ^46,54,58^. The process releases bicarbonate (causing increase in alkalinity) and hydrogen sulfide.

The δ^13^C measurements in this study are based on well-preserved pristine-looking tests. It is nevertheless conceivable that some tests could have had invisible coatings on their inside affecting the original δ^13^C values of the living foraminifera. Given the low δ^13^C we obtained in the calcareous nodules (Fig. 3h; Fig. S4c, e), it is possible that even a thin coating could affect some of the measured foraminiferal values. The δ^18^O values are elevated in the nodules compared to the benthic foraminiferal values but we note that our measured maximal δ^18^O values are similar to and not higher than the values from the non-seep core 1252 (Figs. 4d and 6a; Fig. S4a, d) and other Nordic Seas non-seep records^2,3,6,7,34^. This shows that the thin coatings have not affected the oxygen isotope values in any significant way. We also point out that if the δ^13^C values of the foraminifera were dominated by values from authigenic carbonates precipitated at a migrating SMTZ level, we would expect a random or continuous distribution of the δ^13^C values and not the higher values in stadials and Heinrich stadials, or the correlation to the DO climatic events we see (Figs. 4, 5c and 6b).

The validity of the δ^13^C curve of core 940 as reflecting changes in the emission of methane is supported by several other studies from the Nordic Seas. In core PS62/015 -3 from a methane seep at 980 m water depth in Denmark Strait, Millo et al.^32^ observed a similar stratigraphic distribution of low δ^13^C values (Fig. 1a). In a section referred to MIS 3, the researchers found low δ^13^C in the major interstadials of IS17 to IS8 in a pattern similar to ours and in the long-lasting Heinrich stadials and stadials of MIS 5 and MIS 3, the methane was released before the end of the events similar to H5, S12 and H1 in core 940. In shorter-lasting events the increase was limited to the subsequent interstadial phases^32^ (Fig. 5). Moreover, a comparable distribution of δ^13^C values occurs in Heinrich stadials H4, H3 and H2 in core HH12-930GC from the active pockmark field at 1,200 m water depth on the eastern part of Vestnesa Ridge^47^ (Fig. 1b).

It is also important to notice that core 940 is characterized by many carbonate nodules which mostly occur in the interstadials (but also in H5 and S8–S6). The presence of nodules indicates that methane oxidizing and sulfate reducing microbes were active^46^ (Fig. 3h, Fig. S4c). The very low δ^13^C values signal a strong discharge of methane^59^. The associated high δ^18^O values of the nodules (~ 6 to > 7‰) are a sign of dissociation and thus the presence of gas hydrates^60–62^ (Fig. S4d, e). In periods with strong seepage, nodules tend to form near or at the sediment surface, although they at low seepage also can form deeper in the sediment but at a low rate^58,60,63,64^. In multicore MUC12 from the eastern area of active pockmarks at Vestnesa Ridge in a location of strong seepage and presence of bacterial mats, the SMTZ was located 0–1 cm below the seabed^52^ (Fig. 1b). Total alkalinity was at maximum at 0 cm. The rates of sulfate reduction were at maximum at 0–3 cm below the seabed and methane oxidation rate was at maximum at 3 cm depth.

Additional evidence on changes in the environmental conditions during the investigated period are found in the chemosymbiotic bivalves. They rely on hydrogen sulfide for metabolism. They occur mainly in areas of low to moderate seepage and are absent in areas with intense seepage and toxic levels of sulfide^65,66^. In core 940, they are recorded only in layers correlating with the major stadials and Heinrich stadials of δ^13^C > – 4 to –3‰ and being absent in the interstadials (Figs. 4c and 6b; Fig. S4c, e). This is in good agreement with the interpretations presented above of modest seepage during stadials and Heinrich stadials and strong seepage during interstadials.

### Timing, and magnitude of increased seepage

In this investigation we have shown that during the period 50–12 ka, the highest emissions of methane occurred when the bottom water was coldest (< 0–2 °C), while the lowest occurred when the bottom water was warmest (3–>5 °C). This is opposite to nearly all modern observations from shallow sites at the edge of the GHSZ which find that there is an almost immediate positive correlation between water temperature and the dissociation of gas from gas hydrates^13,15,21,22,29–31^. However, a delayed increase in methane emission agrees with model experiments from deepwater sites which indicate that up to several thousands of years can pass from warming to increased gas release^13,14,67^.

The stadials in the Nordic Seas are distinguished by increasing bottom water temperatures, surface water stratification and a dense cover of sea ice^68^. However, the seepage of gas remained low to moderate until the very end of the stadials signifying that the higher temperatures first at that point in time reached deep, high concentrations of gas hydrates. In terms of years, the delayed reaction relative to the time of the first rise in water temperatures until temperatures begin to decrease is in the order of 500 to > 1,000 years (i.e. the approximate duration of the stadial in the ice cores^8^). The warm bottom water is at the beginning of the interstadials rapidly replaced by cold bottom water due to the abrupt onset of deep convection^3,5–7,34,35^ (Fig. 5a, b, d, e). The response in gas emission to the cooling is delayed with a time factor that resembles the delayed response to the warmings at the beginning of the stadials (Fig. 5c).

The deposits of the Bølling-Allerød interstadials are disturbed from 520 to 260 cm with obliterated sediment structures, abundant pyrite, and a dominance of poorly preserved and apparently reworked foraminifera (Fig. 3h; Fig. S2c; see “Methods”). The reworked faunas include many large, abraded specimens of *C. wuellerstorfi* (Fig. 3f). The most likely cause for these disturbances is turbulence instigated by an intense emission of methane. Upward gas migration is also indicated by the vertical channels in the sediment and by the chaotic isotope values and a fauna distribution pattern with many horizons totally without foraminifera (Fig. 3; Figs. S1, S2). A possible analogue situation could be a human-induced blowout crater from a gas pipe outburst in the North Sea, where small, dead foraminiferal shells were brought in suspension and removed from the area resulting in a local enrichment of large specimens^55^. An extraordinary strong seepage during the Bølling-Allerød interstadials is not surprising considering that the preceding Heinrich stadial H1 experienced the highest bottom water temperatures recorded in the history of core 940 (Figs. 5b and e and 7a and b). Widespread occurrences of low δ^13^C values during the Bølling-Allerød interstadials indicate that similar emissions occurred simultaneously over large areas of the western Svalbard margin^42,47^ including shelf areas^28^. Other contributory causes to the widespread and strong emission of methane during the Bølling and Allerød interstadials could be decreased pressure due to tectonic movements/isostatic rebound^39,47,70–72^, and/or high sedimentation rates^33^. However, the fact that the changes in gas seepage during MIS 2 and MIS 3 occurs on a millennial time scale throughout, suggests that the primary trigger most likely was the high BWT. Gas hydrates are highly sensitive to temperature changes compared to pressure changes^12,14,30^. Tectonism/isostatic rebound will have only a small effect of changes in hydrostatic pressure at this water depth. Furthermore, during MIS 3 the surrounding ice sheets were small and mainly located inland^73^ and the millennial-scale events were probably too short to induce substantial effects from tectonism/isostatic rebounds. Also, sea level changes were small during DO events^74^.

The results of core 940 indicate that the increase in seepage in interstadials is caused by a time lag that can have several causes such as a delay in e.g., slow diffusion rates of heat, the transport rate of gas in fluids or gas phase through the sea bottom sediments depending on porosity and degree of fracturing, and higher microbial activity under warm conditions^14,67^. There are no published diffusion rates from Vestnesa Ridge, but a heat-flow model by Karstens et al.^33^ for the last deglaciation from 600 to 800 m water depth at Nyegga pockmark area (Fig. 1a) indicated a delay of 1,000 years before heat induced by increased sedimentation rates (> 10 m/ka) reached the gas hydrates and an almost explosive seepage of methane occurred.

### Implications

We have studied variations in methane emission at a site from 1294 m water depth at Vestnesa Ridge northwest of Svalbard for the period ~ 50.0–6.3 ka. We find that the emission fluctuates strongly responding to changes in bottom water temperatures, which are linked to the millennial timescale DO events in the Greenland ice cores. However, the increase in emission is delayed with up to > 1,000 years as compared to the temperature increases. We interpret this delay as caused primarily by a slow diffusion of heat through the sediments combined with other subsurface processes acting on release of methane such as the concentration of gas hydrates and the efficiency of the microbial filter. The increase in BWT during the millennial scale events ranges from 1.2 to 4.7 °C with an average increase of 2 °C/ka (Fig. 7). The ocean today has taken up 90% of the atmospheric heating from the present global warming (see references in ref. 24) and the deep ocean is rapidly warming^24,75,76^. Deep water at 700–2,000 m water depth is predicted to warm by 0.2 °C over the next 50 years^76^ which corresponds to 4 °C/ka. This is twice the calculated average rate for the interstadial/stadial (Heinrich stadial) transitions in cores 940 and 1252 (Fig. 7).

At Vestnesa Ridge, bottom water warmings with temperature rises of ~ 3 to > 5 °C continued for 500 to > 1,000 years before the emission of methane started to increase and reached a level sufficient for the formation of authigenic carbonate. Transferred to modern conditions at the site, this would suggest that the present warming of the deep oceans could reach similar temperatures and remain warm for several hundreds of years before we would see any significant reaction in the dissociation of the gas from the buried gas hydrates. However, it also implies that the emission would continue for many years after a stop in the discharge of anthropogenic CO_2_ and a subsequent temperature fall.

## Methods

### Core handling and logging

Piston core HH12-940PC was taken from a sediment-filled pockmark at 1,294 m water depth from the western field of inactive pockmarks on Vestnesa Ridge, northwestern Svalbard margin during a cruise with RV *Helmer Hanssen* in July 2012. The upper part of the 12 m inner core liner imploded at the top and the upper ~ 1 m of sediment was severely disturbed and discarded. The lower 838 cm were intact and appeared undisturbed. The sediments consisted mainly of fine-grained hemipelagic mud and glacimarine coarse, or clayey deposits. However, the interval 545 to 260 cm showed several vertical channels or fractures, and the interval 520 to 260 cm had foraminifera barren intervals and reworked, abraded benthic specimens were occasionally observed (Figs. S1, S2).

Core HH12-940PC was logged using a GEOTEK Multisensor Core Logger with a mounted loop sensor for magnetic susceptibility before opening at the Department of Geosciences, UiT the Arctic University of Norway, Tromsø, Norway (Fig. S2a). The core sections were X-rayed on a GEOTEK Standard X-ray CT System (Fig. S1). After opening the sections were color imaged with a Jai L-107CC 3 CCD RGB line scan camera installed on an Avaatech XRF core scanner (Fig. S1). The lithological log was obtained based on the GEOTEK logs and X-ray images in combination with sediment visual description and color (Munsell chart) shortly after splitting the sections, and the grain size distribution and counts of ice rafted debris (IRD) (see below).

### Sampling and counts of foraminifera and IRD

The entire core was initially sliced in 1-cm-thick samples, weighed, freeze-dried, and weighed again. Samples at 5 cm intervals were taken out and subsequently wet sieved over mesh-sizes 0.063, 0.1, and 0.5 mm. A second round to double the sample resolution to 2.5 cm sample intervals was later done in the lower part > 545 cm. In the interval 570–555 cm samples at every cm were taken out. All new samples were treated in the same way as the first sample batch.

All unsplit sample residues > 0.1 mm was spread evenly on a picking tray. Foraminifera were picked from randomly chosen squares until > 300 specimens of benthic and > 300 specimens of planktic foraminifera were obtained. The foraminifera were identified to species level (some samples contained too few specimens for quantification). The percentages of benthic and planktic species were calculated separately. The size fraction > 0.1 mm was chosen to obtain both small phytodetritus species like *Epistominella* and *Nonionella* species and larger important paleoenvironmental indicator species like e.g., *Melonis barleeanus*, and *Cibicidoides wuellerstorfi* (see species list in Table S1). A total of eight planktic species and > 100 benthic species were identified.

Benthic foraminiferal species of subpolar to subtropical affinity e.g., *Sigmoilopsis schlumbergeri*, *Discospirina italica*, *Opththalmidium inconstans*, *Eggerella bradyi*, *Tosaia hanzawaia*, *Cibicidoides pachyderma*, *Epistominella decorata*, *Gyroidinoides neosoldanii*, and *Gyroidina umbonata* belonging to an association of subtropical to boreal species and defined as the Atlantic Species Group were added together^3,6,7,34–36^ (Table S1). The concentration of planktic and benthic specimens was calculated as number/g dry weight (dwt) sediment (Fig. S2d, e). Ice rafted debris (IRD) were counted in the > 0.5 mm size fraction. Number of IRD grains per g dwt sediments was calculated (Fig. S2b). Carbonate-encrustations (nodules) from authigenic precipitation of carbonates from methane seepage and pyrite particles were also counted in the > 0.5 mm size fraction and concentrations calculated (Fig. 3h, Fig. S2c).

### Transfer functions on the benthic foraminiferal fauna

In core 940 bottom water temperatures (in °C) were estimated by transfer functions using the C2 program^77^. The calculations were based on the > 0.1 mm size fraction of benthic foraminifera. We applied the MAT (Modern Analogue Technique) method using 30 analogues. For the calculations we used a database of 401 samples modified from ref. ^34^ (see references therein). The Root Mean Square Error of Prediction (RMSEP) equals ± 1.8767 °C. The material consists of previously published records on the distribution of benthic foraminifera in the Nordic Seas and in the northern North Atlantic Ocean. For the calculations we used only samples from the depth interval 250 to ~ 2,000 m.

### Macrofaunal counts

Presence and quantification of macrofaunas, large foraminifera and pteropods were examined from the > 0.5 mm size fractions. Chemosymbiotic bivalve species of *Archivesica arctica*, *Isorropodon nyeggaensis*, *Rhagothyas kolgae* and *Acharax svalbardensis* (see Fig. 3; Fig. S2, S4c) were found in distinct thin layers, while others such as rissoid gastropods, other gastropods, *Yoldiella* spp. and *Thyasira* spp. were found more scattered in the record. Large bivalves were often broken but hinges were mostly intact and counted as representative of a shell, because eventual paired (but later separated) shells were not easy to match based on hinges alone. Paired shells of *Yoldiella* spp. and *Thyasira* spp. were common but counted as two to match with other un-paired shell counts.

### Stable isotopes

In core HH12-940PC, stable isotopes δ^18^O and δ^13^C were measured in the planktic foraminiferal species *Neogloboquadrina pachyderma*, and benthic species *Cibibidoides wuellerstorfi*, *Melonis barleeanus*, and *Cassidulina neoteretis*. Only pristine-looking specimens with no visible coating (authigenic precipitation of carbonate; see text for explanation) were picked under a binocular microscope. Several samples in the mid-Sects. 555–270 cm contained few benthic and planktic specimens and several foraminiferal specimens were poorly preserved, therefore in this part benthic isotope analyses were few. Also, deeper in the core some samples were devoid of pristine benthic specimens leaving gaps in the records (marked by black vertical bars in Fig. S4e).

The specimens from the first sample set collected at 5 cm intervals were analyzed using a Kiel IV-MAT 253 at the Department of Earth Science, University of Bergen, Bergen, Norway. Long-term external precision (1σ error), based on the replication of working standards pooled over a period of weeks to months, is ≤ 0.04‰ and ≤ 0.08‰ for δ^13^C and δ^18^O, respectively. Results are reported on the VPDB (Vienna Pee Dee Belemnite) scale and referenced to this scale using NBS-19, NBS-18, and internal house standard CM12 (Table S2).

The samples from the second sample set were analyzed using a ThermoScientific Gasbench II, MAT 253 IRMS at the Department of Geosciences, UiT the Arctic University of Norway, Tromsø, Norway. The precision of the instrument with 1σ error is < 0.1 for both δ^13^C and δ^18^O on calcite. Three standards were used: Isolab A, Isolab B, and Merck CaCO_3_ (Table S2), each reported on the VPDB scale relative to NBS-18, NBS-19 and LSVEC (Table S2).

Because of isotopic disequilibrium, the δ^18^O values of *C. wuellerstorfi* was corrected by + 0.64‰^78^ and *M. barleeanus* by + 0.4‰^79^. The δ^18^O values of the benthic species *C. neoteretis* and the planktic species *N. pachyderma* were not corrected.

Boxplots of isotope data (Fig. 6) were constructed using the Past3 statistical software^80^.

### Calculations of bottom water temperature increases and rate of increases

For both cores the calculation of temperature increases were based on the δ^18^O values by taking the difference between the maximum δ^18^O values at the end of an interstadial and the minimum values obtained in the subsequent stadial/Heinrich stadial. The calculated differences were corrected for the ice volume change using the sea level curve of ref. 74. For core HH15-1252PC we also calculated the temperature differences between the interstadials and stadial/Heinrich stadials using the minimum Mg/Ca temperature and maximum Mg/Ca temperature for each DO event. We refrained from using the temperatures calculated by transfer functions in core HH12-940PC. In the Svalbard region, the calculations based on benthic species seem to slightly overestimate the lowest temperatures between − 1 and 1 °C^34^. The rates of temperature increases were calculated for each event by dividing the calculated ranges of temperature rises by the time of minimum temperature to the maximum based on the NGRIP ice core age model^8^. The errors of the duration of stadials and Heinrich stadials in the ice core are very small ranging from ~ 75 years in stadials S7 and S8 both lasting about 1,000 years, to 225 years in Heinrich Stadial H2 lasting about 4,200 years (Table S3). In percentage the error range from 2.6% during H3 to 7.3% during GS7. The range of the calculated temperature increases may be underestimated because we may not have resolved the full range because of low time resolution in some events.

### AMS-14C dates

In core HH12-940PC, samples of pristine and empty shells of monospecific *N. pachyderma*, and shells of chemosymbiotic bivalves, and small non-chemosymbiotic bivalves were dated (Table 1). In two cases, the levels dated by *N. pachyderma* were also dated using chemosymbiotic bivalves. Mixed planktic and benthic foraminiferal faunas were dated in intervals where dateable material was scarce, sometimes supplemented by small specimens of non-chemosymbiotic bivalves (Table 1). We allow these datings based on mixed benthic and planktic material, because there is only a small difference in reservoir effect between surface and bottom in the Nordic Seas^3,6–11,34,81^.

The AMS^14^C dates were performed at the 14Chrono Centre, Queen’s University, Belfast, Northern Ireland, UK. The AMS-laboratory routinely acid etch samples to remove contaminants on inner and outer shell surfaces to run only presumably uncontaminated inner parts of shells. To calibrate the radiocarbon ages, we used the approach presented in Heaton et al.^81^ which accounts for some latitudinal and regional uncertainties related to^14^C depletion. We extracted the average regional marine radiocarbon reservoir age (ΔR) from the http://calib.org/marine/ database based on the seven nearest modern-day ΔR. This resulted in a ΔR = – 65 ± 33 ^14^C years for our study area (Table 1). Individual samples were then calibrated using the IntCal 0.3.1 package (https://cran.rproject.org/web/packages/IntCal/IntCal.pdf).

We used Bayesian age-depth modelling (Table 1), the Marine20 calibration curve^81^ and the rBacon 3.1.1 package^82^ in R software for the age-depth plot (Fig. 2a). The ages in this paper are presented using the modern ΔR. The mid-points of obtained age ranges were chosen, and calibrated ages are reported with 1σ error (Fig. 2a; Table 1). Two dates are clearly outliers. A date from 550.5 cm performed on *N. pachyderma* was > 7,000 years older than the date from the same level performed on *Thyasira* sp. (Fig. 2; Table S1) and we suspect that the specimens were contaminated by old carbonate from seepage of gas^48^. The same can be true for the sample from 657.5 cm.

To test our identification of individual DO events and their subdivision into stadials/Heinrich stadials and interstadials, we correlated the events in core 940 to the events in nearby non-seep core 1252 which was tied to the δ^18^O record of the NGRIP ice core^7^ (Figs. 2b and 5; Figs. S3, S4). While the identification of the individual DO events in the two marine cores appear straightforward, it is evident that there is a divergence between the calibrated ages of the events in the marine cores and their ages in the NGRIP time scale^7^. This time difference amounts of > ~ 1,000 years in MIS 2 and up to 2,000 years in parts of MIS 3. The difference in calibrated years and the ice core time scale is likely reflecting an increase in the reservoir age during the glacial although contamination cannot be excluded. For instance, ΔR in the surface water increased to 2,000 years during H4 in a non-seep record from the southern Norwegian Sea^83^ (Fig. 2b). We refrain from plotting our data on an age scale as the profound changes in sedimentation rates stretches some intervals while compressing others making figures difficult to overview (Fig. 2b, c).

## Electronic supplementary material

Below is the link to the electronic supplementary material.

Supplementary Material 1

## Data Availability

The data is available at DataverseNO, the UiT Open Research Data repository: https://doi.org/10.18710/QLMJVH.
