# Supplementary material for: Variations in deep-sea methane seepage linked to millennial-scale changes in bottom water temperatures ~ 50–6 ka, NW Svalbard margin
Source: Sci Rep. 2024 Sep 27;14:22184. doi: 10.1038/s41598-024-72865-3 (PMC11436790; doi:10.1038/s41598-024-72865-3)
Supplement: Supplementary file 1 — Supplementary Material 1 [file 41598_2024_72865_MOESM1_ESM.pdf]

Supplementary information for:

Variations in deep-sea methane seepage linked to millennial-scale changes in bottom water temperatures ~50–6 ka, NW Svalbard margin

Tine L. Rasmussen[1]*, Naima El bani Altuna[1], and Erik Thomsen[2]
[1]Department of Geosciences, UiT – The Arctic University of Norway, Tromsø, 9010, Norway
[2]Department of Geosciences, Aarhus University; Aarhus 8000, Denmark

Content

The supplementary information provides additional figures, tables, and interpretations.

- Figs. S1-S4
- Tables S1-S2
- Lithology, magnetic susceptibility, mineral content, planktic and benthic foraminiferal records and chemosymbiotic marcofaunas in core 940
- Interpretation of paleoenvironments in core 940 and 1252 from benthic foraminiferal faunas

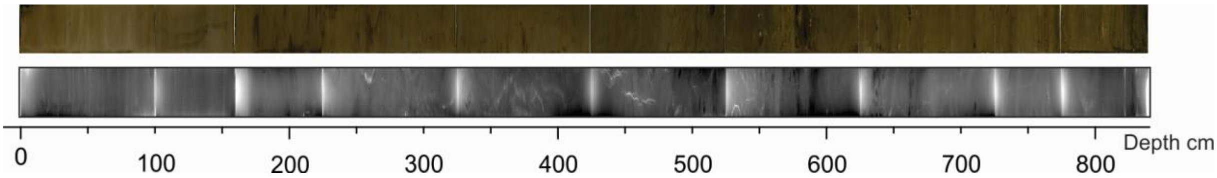

Fig. S1. **X-ray and XRF image scan of core HH12-940PC**. Top: XRF image scan, below: X-ray photos and depth scale. Note that the depth scale of these do not completely fit the analyzed sample depths. The core measured 846 cm when retrieved and logged by GEOTEK. After slicing for samples at 1-cm intervals, the core measured 838 cm indicating some samples may have been thicker than 1 cm.

Lithology, magnetic susceptibility, mineral content, planktic and benthic foraminiferal records, and chemosymbiotic macrofaunas. Core 940 shows several distinct lithological features that clearly have stratigraphical value as they occur in numerous other records from the western Svalbard margin and always at the same stratigraphical position (Jessen et al., 2010; Rasmussen et al., 2014; Sztybor and Rasmussen, 2017; El bani Altuna et al., 2021; Rasmussen and Nielsen, 2024) (Fig. S2). These features comprise a dark grey-brown, coarse, unsorted debris flow/IRD deposit from the last glacial maximum dating ~24.0 ka (582.5–575.5 cm down core), a clayey, blueish laminated horizon deposited during the Bølling interstadial dating ~14.6 ka (430–480 cm down core), and an early Holocene diatom layer dating ~10.0 ka (175–180 cm down core) (Fig. S2).

The magnetic susceptibility values in core 940 are very low and constant except in the uppermost part. This is due to degradation of magnetic particles caused by seepage of methane and typical for sites with a strong influence of seepage (Sztybor and Rasmussen, 2017 and references therein). The concentration of IRD is very variable in MIS 3 typical for numerous records from the western Svalbard margin. The maximum is in MIS 2 and minimum in the Holocene (e.g., Jessen et al., 2010 and references therein; Jessen and Rasmussen, 2019). The highest concentration of calcareous nodules from precipitation of authigenic carbonates (see main text for explanation and discussion) is found in MIS 3 and in the coarse unsorted layer in MIS 2. The concentration of pyrite particles is high during the main part of the deglaciation (Bølling and Allerød interstadials into the lower Holocene).

The concentrations of planktic and benthic foraminifera show patterns typical of the western Svalbard margin with very variable concentrations and variable productivity in MIS 3, and high concentrations in peak MIS 2. The concentration during the Bølling and Allerød interstadials is at minimum. It is high in the Holocene interglacial indicating increased productivity (e.g., Rasmussen et al., 2007; Rasmussen et al., 2014).

The most common chemosymbiotic species in core 940 are *Archivesica arctica*, and *Isorropodon nyeggaensis*, accompanied by the assumed chemosymbiotic species *Rhagothyas kolgae* and rare *Acharax svalbardensis*. They have also been found in cores from the eastern part of Vestnesa Ridge (Åström et al., 2017; Hansen et al., 2017, 2020; Thomsen et al., 2019) and in seep areas in Nyegga, Vøring Plateau (Krylova et al., 2011). These four species co-occur with epifaunal rissoan gastropods of species *Frigidoalvania* spp. and naticids (Fig. 4c). In the older part, the shells are corroded and fragmented, but fragments of the more robust

species *A. arctica* and *R. kolgae* are still identifiable (Fig. S2). One single specimen of *I. nyeggaensis* is found in sediments of Younger Dryas age (i.e., = stadial S1) (Fig. 3, Fig. S2).

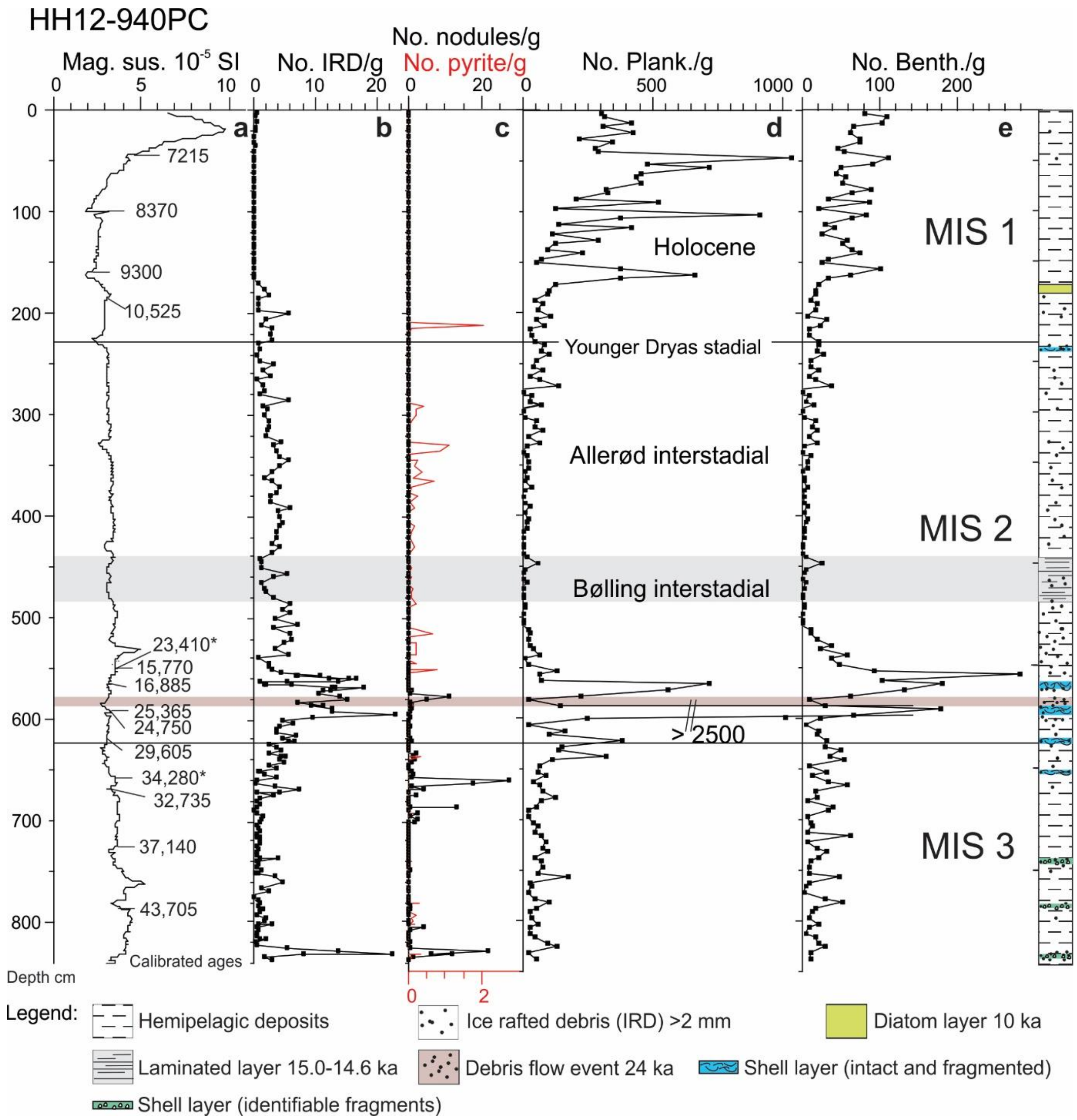

Fig. S2. **Magnetic susceptibility and other data from core HH12-940PC**. **a** Magnetic susceptibility in 10^{-5} SI units. Calibrated ages are indicated, asterisks mark outlier dates. **b** Concentration of ice rafted debris (IRD) >0.5 mm in number per gram dry weight sediment. **c** Concentration of calcareous nodules >0.5 mm from authigenic carbonate precipitation (see text for explanation) in number per gram dry weight sediment (black curve) and concentration of pyrite particles >0.5 mm in number per gram dry weight sediment (red curve). **d** Concentration of planktic foraminifera in number per gram dry weight sediment. **e**

Concentration of benthic foraminifera in number per gram dry weight sediment. Lithological log is shown to the right. Marine isotope stages (MIS) and boundaries are indicated.

Interpretation of paleoenvironments in core 940 and 1252 from benthic foraminiferal faunas. Overall, in core 1252 and core 940 the benthic foraminiferal assemblages during MIS 3 and MIS 2 are dominated by *Cassidulina neoteretis*, *Melonis barleeanus*, *Cassidulina reniforme*, *Stainforthia loeblichi*, and *Nonionella* spp. (Fig. S3). In both records, interstadials are characterized by similarly high benthic $\delta^{18}O$, low BWT and presence/dominance by *M. barleeanus, C. reniforme*, *Stainforthia* spp. (in core 940 mainly *Stainforthia loeblichi*), and *Nonionella* spp. (Fig. S3). In core 940, *Nonionella* spp. comprise mainly the species *N. turgida*, *N. iridea* and *N. stella*, being generally opportunistic and indicative of short-lasting seasonally high productivity (Sejrup at al., 2004; Gooday and Hughes, 2002; Rasmussen and Thomsen, 2017 and references therein; El bani Altuna et al., 2021 and references therein). *Stainforthia loeblichi* is an indicator of presence of seasonal sea ice (e.g., Seidenkrantz, 2013) (Fig. S3). Together with *Nonionella* spp. it tends to appear early in the interstadials probably indicating presence of sea ice and short seasonal productivity as land ice is retreating and sedimentation rates are high (e.g., Rasmussen and Thomsen, 2013; Jessen and Rasmussen, 2019). *Cassidulina reniforme* is more consistently present in the interstadials. It is an indicator of chilled Atlantic Water and bottom currents (Steinsund, 1994). *Melonis barleeanus* is generally most abundant late in the interstadial, indicating higher food production and more stable conditions and less sea ice (e.g., Mackensen, 1987). All-in-all, the interstadial species together with BWT and $\delta^{18}O$ values indicate ‘interglacial-like’ conditions with convection in the Nordic Seas in line with previous results from the Nordic Seas and North Atlantic (e.g., Rasmussen et al., 1996; Rasmussen and Thomsen, 2004; Ezat et al., 2014; El bani Altuna et al., 2021).

In cores 940 and 1252, stadials and Heinrich stadials show low benthic $\delta^{18}O$, high BWT and benthic faunas that are dominated by *C. neoteretis*, and Atlantic species, a group comprised of warm water species, being most abundant in Heinrich stadial H1 (Chauhan et al., 2016; Ezat et al., 2014; El bani Altuna et al., 2021) (Fig. S3). *Cassidulina neoteretis* is mostly found below stratified surface water conditions with extensive sea-ice cover (e.g., Jennings and Helgadottir, 1994; Lubinski et al., 2001: Cage et al., 2021; El bani Altuna et al., 2021 and references therein) and is typically dominant in stadial and Heinrich stadials in the Nordic Seas, northern North Atlantic and Arctic Ocean when warm Atlantic Water circulates

as an intermediate water mass (e.g., Rasmussen et al., 1996; Rasmussen and Thomsen, 2004, 2013; Marcott et al., 2011; Chauhan et al., 2016; El bani Altuna et al., 2021).

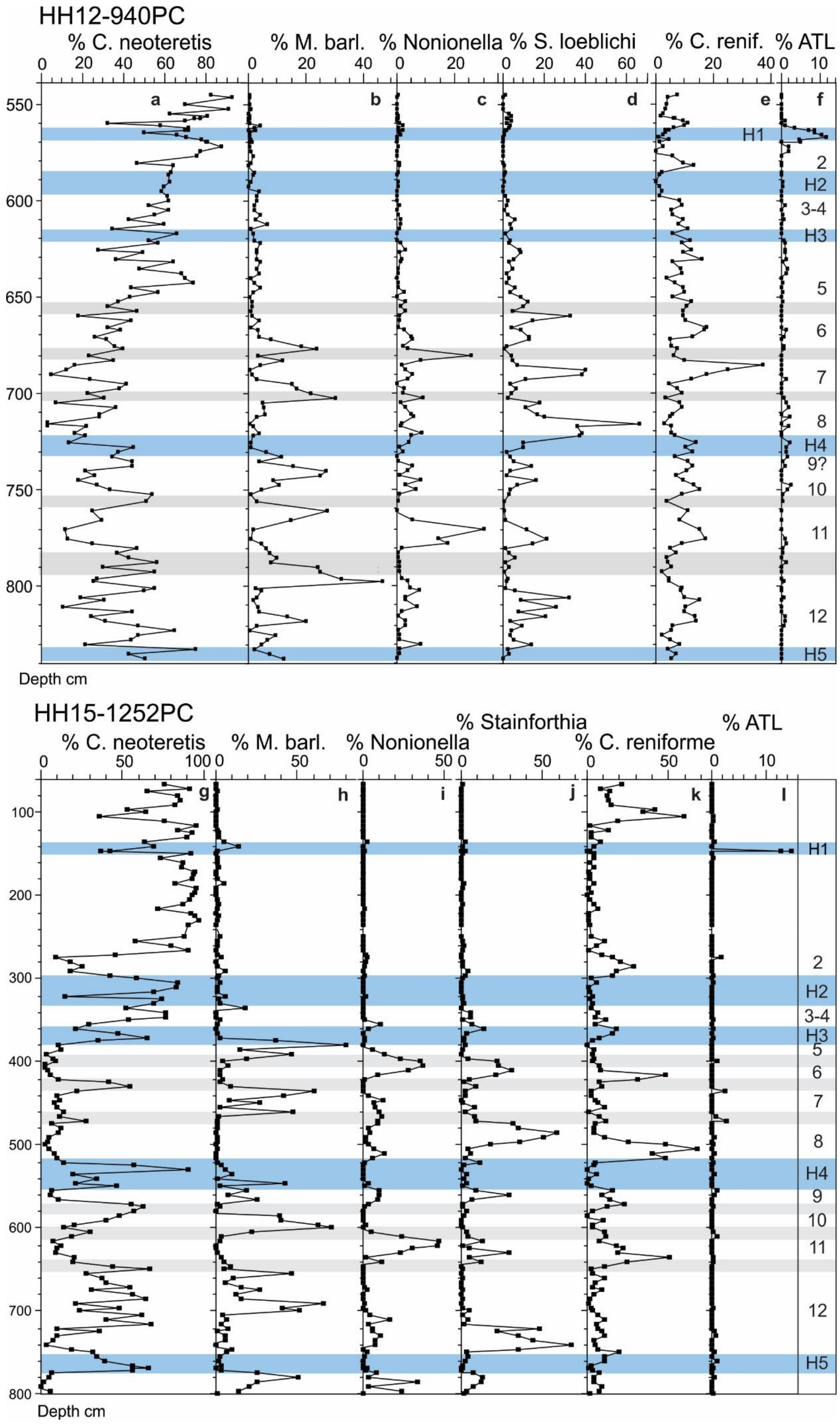
HH12-940PC
% C. neoteretis
% M. barl.
% Nonionella
% S. loeblichi
% C. renif.
% ATL
Depth cm
H1
2
H2
3-4
H3
5
6
7
8
H4
9?
10
11
12
H5
HH15-1252PC
% C. neoteretis
% M. barl.
% Nonionella
% Stainforthia
% C. reniforme
% ATL
Depth cm

Fig. S3. Distribution of selected benthic foraminiferal species for cores HH12-940PC (a–f, this study) and HH15-1252PC (g–l; El bani Altuna et al., 2021). **a** Percentage of benthic foraminiferal species *Cassidulina neoteretis*. **b** Percentage of *Melonis barleeanus*. **c** Percentage *Nonionella* spp. **d** Percentage of *Stainforthia loeblichi*. **e** Percentage of *Cassidulina reniforme*. **f** Percentage of Atlantic species group (ATL). Heinrich events H5 to H1 and interstadial numbers 12–2 are indicated. **g–l** Distribution of same species in core HH15-1252PC (data from El bani Altuna et al., 2021). Heinrich stadials H5 to H1 and stadial (S) and interstadial numbers 12–2 are indicated. Blue horizontal bars mark Heinrich stadials, grey bars mark stadials.

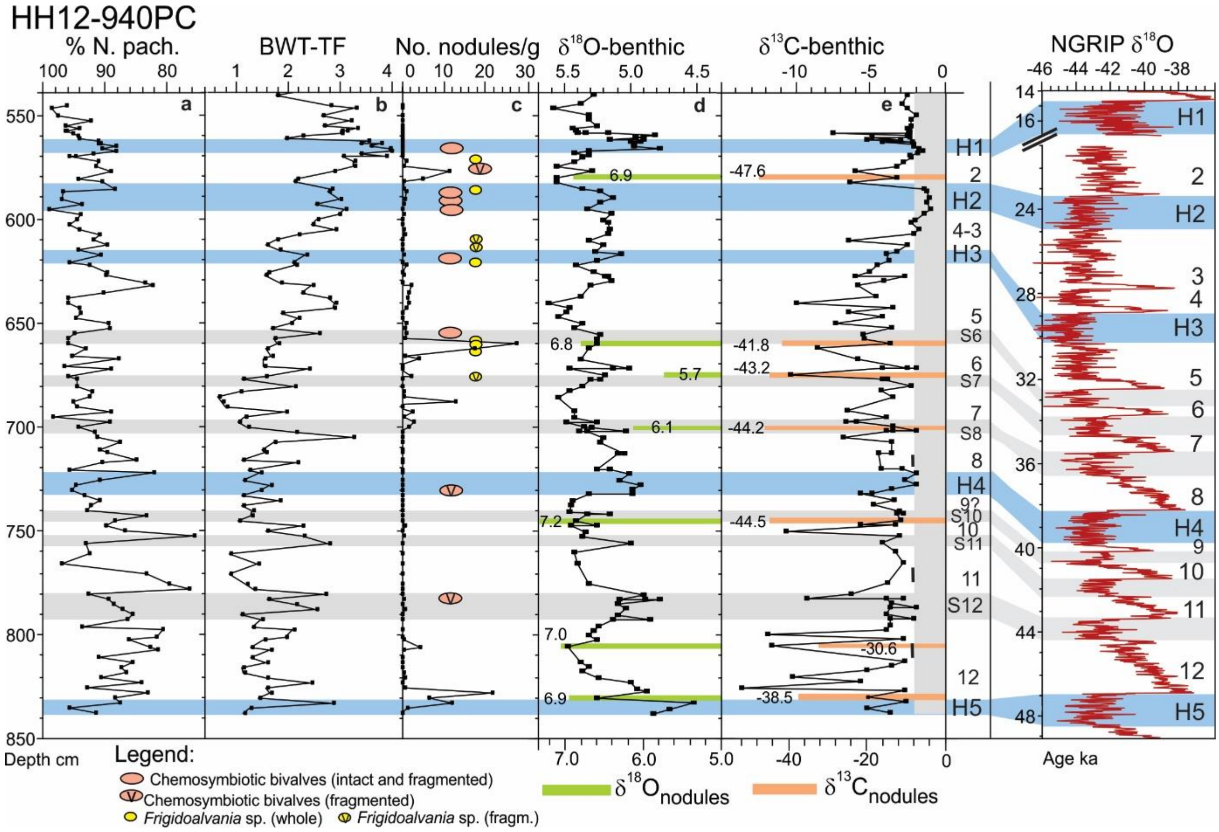

Fig. S4. Details in various paramters in core HH12-940PC for the lower 545–838 cm (MIS 3 and MIS 2). Percentage distribution of polar planktic foraminiferal species *Neogloboquadrina pachyderma* (N. pach.; note inverse scale). These changes in relative abundance of *N. pachyderma* are typical for the Nordic Seas where it reflects shifts in influence of cold polar surface water, and warm Atlantic water (e.g., Bond et al., 1993; Rasmussen and Thomsen, 2004). **b** Absolute bottom water temperatures (BWT) calculated from transfer functions (TF) of the benthic foraminiferal faunas in the core. **c** Concentration

of calcareous nodules >0.5 mm from authigenic carbonate precipitation (see text for explanation) in number per gram dry weight sediment. Position of layers of chemosymbiotic bivalves and gastropods are indicated (see legend below). **d** Corrected benthic $\delta^{18}O$ measured in *Melonis barleeanus* and *Cassidulina neoteretis,* and $\delta^{18}O$ measured in authigenic carbonate nodules with values indicated (green bars) (see text for explanation). **e** Benthic $\delta^{13}C$ measured in *M. barleeanus* and *C. neoteretis*. Vertical grey bar indicates typical range of glacial and interglacial values. Light orange bars are $\delta^{13}C$ measured in authigenic carbonate nodules (see text for explanation) with values indicated. Vertical black bars show intervals with too few pristine shells for isotope analysis. To the right are shown NGRIP Ice Core $\delta^{18}O$ data versus SMOW[14]. Note break in y-axis for the LGM interval 21–17 ka. Heinrich stadials H5 to H1, stadial (S) and interstadial numbers 12–2 are indicated. Blue horizontal bars mark Heinrich stadials, grey bars stadials. Note few data points in interstadial 8 in core 940, because of too few pristine foraminiferal specimens available for analysis (severe coatings of shells with authigenic carbonate).

Table S1. Species list of planktic and benthic foraminiferal species and taxonomic references. The list contains foraminiferal species in alphabetical order within each foraminiferal group of planktic species, benthic species subgroups ’agglutinated species’, ‘Porcelaneous species’ and ‘Hyaline species’ from core HH12-940PC with references totaxonomy. Asterix marks species of the Atlantic species group.

Planktic foraminifera:
Globigerina bulloides d’Orbigny, 1826
Globigerinita glutinata (Egger, 1893)
Globigerinita uvula (Ehrenberg, 1861)
Globorotalia inflata (d’Orbigny, 1839)
Globorotalia scitula (Brady, 1882)
Neogloboquadrina incompta (Cifelli, 1961)
Neogloboquadrina pachyderma (Ehrenberg, 1861)
Turborotalita quinqueloba (Natland, 1938)

Benthic foraminifera:
Benthic species marked by an asterisk were analyzed as a group of Atlantic species in the transfer function calculations.

Agglutinated species:

Adercotryma glomerata (Brady, 1878)
Ammodiscus spp.
Cribrostomoides jeffreysii (Williamson, 1858)
Cribrostomoides subglobosa (Sars, 1910)
Deuterammina montagui Brönnimann and Whittaker, 1988
Deuterammina ochracea (Williamson, 1858)
Eggerella bradyi (Cushman, 1911)*
Eggerella advena (Cushman, 1922)
Hyperammina spp.
Lagenammina difflugiformis (Brady, 1879)
Portatrochammina bipolaris Brönnimann and Whittaker, 1980
Recurvoides turbinatus (Brady, 1881)
Reophax scorpiurus Montfort, 1808
Rhabammina abyssorum Sars in Carpenter, 1869
Psammosphaera fusca Schulze, 1875
Siphotextularia rholshauseni Phleger and Parker, 1951
Spiroplectammina biformis (Parker and Jones 1865)
Trochammina globigeriniformis (Parker and Jones, 1865)
Trochammina spp.
Agglutinated indeterminata

Porcelaneous species:
Cornuspira distincta (Cole & Scott 2008)
Cornuspira foliacea (Philippi, 1844)
Cyclogyra involvens (Reuss, 1850)
Discospirina italica (Costa, 1856)*
Glomulina oculus Jennings, Seidenkrantz and Knudsen, 2020
Miliolinella subrotunda (Montagu, 1803)
Nummoloculina irregularis (d'Orbigny, 1839)*
Ophthalmidium inconstans (Brady, 1879)*
Pyrgo elongata (d'Orbigny, 1826)*
Pyrgo oblonga (Montagu, 1803)
Quinqueloculina lamarckiana d'Orbigny, 1839
Quinqueloculina padana Perconig, 1954*
Quinqueloculina seminula (Linnaeus, 1758)
Sigmoilina tenuis (Czjzek, 1848)*
Sigmoilopsis schlumbergeri (Silvestri, 1904)*
Spirophthalmidium acutimargo (Brady, 1884)*
Triloculina tricarinata d'Orbigny, 1826

Hyaline species:
Anomalinoides minimus (Vismara-Schilling and Parisi, 1981)*
Astrononion gallowayi Loeblich and Tappan, 1953
Bolivina pseudopunctata Höglund, 1947

Buccella calida (Cushman & Cole 1930)
Buccella spp.
Bulimina aculeata d'Orbigny, 1826
Bulimina costata d'Orbigny, 1826*
Buliminella elegantissima (d'Orbigny, 1839)
Cassidulina neoteretis Seidenkrantz, 1995
Cassidulina reniforme Nørvang, 1945
Cassidulina spp.
Cibicides lobatulus Walker and Jacob, 1798
Cibicidoides pachyderma (Rzehak, 1886)*
Cibicidoides wuellerstorfi (Schwager, 1866)
Discorbinella berthelothi (d'Orbigny, 1839)
Discorbis williamsoni Chapman and Parr, 1932
Discorbis spp.
Elphidium clavatum Cushman 1930
Elphidium subarcticum Cushman, 1944
Elphidium spp.
Eponides tumidulus (Brady, 1884)
Epistominella arctica Green, 1960
Epistominella decorata (Phleger and Parker, 1951)*
Epistominella exigua (Brady, 1884)
Epistominella vitrea Parker, 1953
Fissurina spp.
Fissurina lagenoides (Williamson, 1858)
Globobulimina auriculata (Bailey, 1851)
Gyroidina lamarckiana (d'Orbigny, 1839)
Gyroidina umbonata (Silvestri, 1898)*
Gyroidinoides neosoldanii Brotzen, 1936*
Islandiella norcrossi (Cushman, 1933)
Islandiella islandica (Nørvang, 1945)
Lagena gracillima (Seguenza, 1862)
Lagena gracilis Williamson, 1848
Lagena gracilis var.
Lagena laevis Montagu, 1803
Lagena nebulosa (Cushman)
Lagena semistriata Williamsson, 1848
Lagena striata (d'Orbigny, 1839)
Lagena striatopunctata Parker and Jones, 1865
Lagena sulcata spicata (Cushman and McCulloch, 1950)
Lamarckina haliotidea (Heron-Allen and Earland, 1911)
Lenticulina spp.
Marginulina costata (Batsch, 1791)
Melonis barleeanus (Williamson, 1858)
Nonionellina labradorica (Dawson, 1860)

Nonionella auricula Heron-Allen and Earland, 1930
Nonionella iridea Heron-Allen and Earland, 1932
Nonionella stella (Cusnman and Moyer, 1930)
Nonionella turgida (Williamson, 1858)
Nonionella opima digitata (Nørvang, 1945)
Nodosaridae
Oolina hexagona (Montagu, 1803)
Oolina melo d'Orbigny, 1839
Oolina spp.
Oridorsalis umbonatus (Reuss, 1851)
Parafissurina spp
Parafissurina staphyllearia (Schwager, 1866)
Parafissurina tectulostoma Loeblich and Tappan, 1953
Patellina corrugata Williamson, 1858
Polymorphinida
Pullenia bulloides (d'Orbigny, 1826)
Pullenia osloensis Feyling-Hanssen, 1964
Pullenia subcarinata d'Orbigny, 1839)*
Robertinoides charlottensis (Cushman, 1925)
Robertinoides spp.
Sagrina subspinescens (Cushman, 1922)*
Stainforthia loeblichi (Feyling-Hanssen, 1954)
Stainforthia sp
Stainforthia feylingi Knudsen and Seidenkrantz, 1993
Tosaia hanzawaia Takayanagi, 1953*
Trifarina angulosa (Williamson, 1858)
Trifarina fluens (Todd, 1948)
Valvulineria arctica Green, 1859

Table S2. Carbon and oxygen isotope values of standards used in stable isotope analysis

**Standard name**	**δ^{13}C-VPDB [‰]**	**δ^{18}O-VPDB [‰]**
CM12	2.10	-1.92
Isolab A	1.96	-2.15
Isolab B	-10.21	-18.59
Merck $CaCO_3$	-48.95	-13.91

Table S3. Start, end, duration and maximum error of duration of Heinrich stadials and stadials measured in GICC05 years (Rasmussen et al., 2014).

Heinrich stadial/ Stadial	Age start	Age end	Duration	Maximum error	Error in %
H5	48,340	46,860	1,480	76	5.1
H4	39,900	38,220	1,689	120	4.8
H3	30,600	28,900	1,700	110	2.6
H2	27,540	23,340	4,200	226	5.4
H1	17,480	14,690	2,510	114	2.7
GS8	36,580	35,480	1,100	76	6.9
GS7	34,740	33,740	1,000	74	7.3

References

Åström, E. K. L., Oliver, P. G. & Carroll, M. L. A new genus and two new species of Thyasiridae associated with methane seeps off Svalbard, Arctic Ocean. *Mar. Biol. Res*. **13**, 402–416 (2017).

Bond, G. *et al*. Correlations between climate records from North Atlantic and Greenland ice. *Nature* **365**, 343–347 (1993).

Cage, A. G., Pieńkowski, A. J., Jennings, A., Knudsen, K. L. & Seidenkrantz, M. -S. Comparative analysis of six common foraminiferal species of the genera *Cassidulina*, *Paracassidulina*, and *Islandiella* from the Arctic-North Atlantic domain. *J. Micropalaeontol*. **40**, 37–60 (2021).

Chauhan, T., Rasmussen, T. L. & Noormets, R. Palaeoceanography of the Barents Sea continental margin, north of Nordaustlandet, Svalbard, during the last 74 ka. *Boreas* **45**, 76–99. https://doi.org/10.1111/bor.121352016 (2016).

El bani Altuna, N., Ezat, M. M., Greaves, M. & Rasmussen, T. L. Millennial-scale changes in bottom water temperature and water mass exchange through the Fram Strait 79ºN, 63–13 ka. *Paleoceanogr. Paleoclimatol*. **36**, e2020PA004061. doi: 10.1029/2020PA004061 (2021).

Ezat, M., Rasmussen, T. L. & Groeneveld, J. Persistent intermediate water warming during cold stadials in the southeastern Nordic seas during the past 65 k.y. *Geology* **42**, 663–666 (2014).

Gooday, A. J. & Hughes, J. A. Foraminifera associated with phytodetritus deposits at a bathyal site in the northern Rockall Trough (NE Atlantic): Seasonal contrasts and a comparison of stained and dead assemblages. *Mar. Micropaleontol*. **46**, 83–110. https://doi.org/10.1016/s0377-8398(02)00050-6 (2002).

Hansen, J., Hoff, U., Sztybor, K. & Rasmussen, T. L. Taxonomy and palaeoecology of two Late Pleistocene species of Vesicomyid bivalves from cold methane seeps at Svalbard 79°N. *J. Molluscan Studies* **3**, 270–279 (2017).

Hansen, J., Ezat, M. M., Åström, E. K. L. & Rasmussen, T. L. New Late Pleistocene species of *Acharax* from Arctic methane seeps off Svalbard. J. *Systematic Palaeontol*. **18**, 197–212 (2020).

Jennings, A. E. & Helgadottir, G. Foraminiferal assemblages from the fjords and shelf of eastern Greenland. *J. Foram. Res*. **24**, 123–144. https://doi.org/10.2113/gsjfr.24.2.123 (1994).

Jessen, S. P. & Rasmussen, T. L. Ice rafting patterns on the western Svalbard slope 74–0 ka: Interplay between ice-sheet activity, climate and ocean circulation. *Boreas* **48**, 236–256 (2019).

Jessen, S. P., Rasmussen, T. L., Nielsen, T. & Solheim, A. A new Late Weichselian and Holocene marine chronology for the western Svalbard slope 30,000–0 cal years BP. *Quat. Sci. Rev*. **29**, 1301–1312 (2010).

Krylova, E. M., Gebruk, A. V., Portnova, D. A., Todt, C. & Haflidason, H. New species of the genus *Isorropodon* (Bivalvia: Vesicomyidae: Pliocardiinae) from cold methane seeps at Nyegga (Norwegian Sea, Vøring Plateau, Storegga Slide). *J. Mar. Biol*. **91**, 1135–1144 (2011).

Lubinski, D. J., Polyak, L. & Forman, S. L. Freshwater and Atlantic water inflows to the deep northern Barents and Kara seas since ca 13 ^{14}C ka: Foraminifera and stable isotopes. *Quat. Sci. Rev*. **20**, 1851–1879. https://doi.org/10.1016/s0277-3791(01)00016-6 (2001).

Mackensen, A. 1987. Bentische Foraminiferen auf dem Island-Schottland Rücken: Umwelt-Anzeiger an der Grenze zweier Ozeansicher Raume. *Paläontol. Z*. **61**, 149–179.

Marcott, S. A. *et al*. Ice-shelf collapse from subsurface warming as a trigger for Heinrich events. PNAS 108, 13415–13419. https://doi.org/10.1073/pnas.1104772108 (2011).

Rasmussen, S. O. *et al*. A stratigraphic framework for abrupt climatic changes during the last glacial period based on three synchronized Greenland ice-core records: refining and extending the INTIMATE event stratigraphy. *Quat. Sci. Rev.* **106**, 14–28 (2014).

Rasmussen, T. L. & Nielsen, T. Glacial-interglacial control on gas seepage exemplified by Vestnesa Ridge off NW Svalbard margin. *Front. Earth Sci*. **12**. doi:10.3389/feart2024.1356341 (2024).

Rasmussen, T. L. & Thomsen, E. The role of the North Atlantic Drift in the millennial timescale glacial climate fluctuations. *Palaeogeogr., Palaeoclim., Palaeoeco*l. **210**, 101–116 (2004).

Rasmussen, T. L. & Thomsen, E. Pink marine sediments reveal rapid ice melt and Arctic meltwater discharge during Dansgaard-Oeschger warmings. *Nat. Comm*. **4**, 2849. doi: 10.1038/ncomms3849 (2013).

Rasmussen, T. L., Thomsen, E. & Nielsen, T. Water mass exchange between the Nordic seas and the Arctic Ocean on millennial time scale during MIS 4–MIS 2. *Geochem., Geophys., Geosys*. **15**, 530–544. doi: 10.1002/2013GC005020 (2014).

Rasmussen, T. L. & Thomsen, E. Ecology of deep-sea benthic foraminifera in the North Atlantic during the last glaciation: food or temperature control. *Palaeogeogr., Palaeoclimatol., Palaeoecol*. **472**, 15–32. doi.org/10.1016/j.palaeo.2017.02.012 (2017).

Rasmussen, T. L., Thomsen, E., Labeyrie, L. & van Weering, T. C. E. Circulation changes in the Faeroe-Shetland Channel correlating with cold events during the last glacial period (58–10 ka). *Geology* **24**, 937–940 (1996).

Rasmussen, T. L. *et al*. Paleoceanographic evolution of the SW Svalbard margin (76°N) since 20,000 ^{14}C yr BP. *Quat. Res*. **67**, 100–114 (2007).

Seidenkrantz, M. S. Benthic foraminifera as palaeo sea-ice indicators in the subarctic realm-examples from the Labrador Sea-Baffin Bay region. *Quat. Sci. Rev*. **79**, 135–144. https://doi.org/10.1016/j.quascirev.2013.03.014 (2013).

Sejrup, H. P., Birks, H. J. B., Klitgaard Kristensen, D. & Madsen, H. Benthonic foraminiferal distributions and quantitative transfer functions for the northwest European continental margin. *Mar. Micropaleontol*. **53**, 197–226 (2004).

Steinsund, P. I. Benthic foraminifera in surface sediments of the Barents and Kara seas: Modern and late Quaternary applications. *PhD Thesis*, UiT the Arctic University of Norway, Tromsø, pp. 1–111 (1994).

Sztybor, K. & Rasmussen, T. L. Diagenetic disturbances of marine sedimentary records from methane influenced environments in the Fram Strait as indications for variation in seep intensity during the last 35 000 years. *Boreas* **46**, 212–228 (2017).

Thomsen, E. *et al*. Cold-seep fossil macrofaunal assemblages from Vestnesa Ridge, eastern Fram Strait during the past 45 000 years. *Polar Res*. **38**, 3310. http://dx.doi.org/10.33265/polar.v38.3310 (2019).
